# CCPA: cloud-based, self-learning modules for consensus pathway analysis using GO, KEGG and Reactome

**DOI:** 10.1093/bib/bbae222

**Published:** 2024-07-23

**Authors:** Ha Nguyen, Van-Dung Pham, Hung Nguyen, Bang Tran, Juli Petereit, Tin Nguyen

**Affiliations:** Department of Computer Science and Software Engineering, Auburn University, AL 36849, USA; Department of Computer Science and Software Engineering, Auburn University, AL 36849, USA; Department of Computer Science and Software Engineering, Auburn University, AL 36849, USA; Department of Computer Science, California State University, Sacramento, CA 95819, USA; Nevada Bioinformatics Center, University of Nevada, Reno, NV 89557, USA; Department of Computer Science and Software Engineering, Auburn University, AL 36849, USA

**Keywords:** cloud computing, self-learning module, NIGMS sandbox, pathway analysis, jupyter notebooks

## Abstract

This manuscript describes the development of a resource module that is part of a learning platform named ‘NIGMS Sandbox for Cloud-based Learning’ (https://github.com/NIGMS/NIGMS-Sandbox). The module delivers learning materials on Cloud-based Consensus Pathway Analysis in an interactive format that uses appropriate cloud resources for data access and analyses. Pathway analysis is important because it allows us to gain insights into biological mechanisms underlying conditions. But the availability of many pathway analysis methods, the requirement of coding skills, and the focus of current tools on only a few species all make it very difficult for biomedical researchers to self-learn and perform pathway analysis efficiently. Furthermore, there is a lack of tools that allow researchers to compare analysis results obtained from different experiments and different analysis methods to find consensus results. To address these challenges, we have designed a cloud-based, self-learning module that provides consensus results among established, state-of-the-art pathway analysis techniques to provide students and researchers with necessary training and example materials. The training module consists of five Jupyter Notebooks that provide complete tutorials for the following tasks: (i) process expression data, (ii) perform differential analysis, visualize and compare the results obtained from four differential analysis methods (limma, t-test, edgeR, DESeq2), (iii) process three pathway databases (GO, KEGG and Reactome), (iv) perform pathway analysis using eight methods (ORA, CAMERA, KS test, Wilcoxon test, FGSEA, GSA, SAFE and PADOG) and (v) combine results of multiple analyses. We also provide examples, source code, explanations and instructional videos for trainees to complete each Jupyter Notebook. The module supports the analysis for many model (e.g. human, mouse, fruit fly, zebra fish) and non-model species. The module is publicly available at https://github.com/NIGMS/Consensus-Pathway-Analysis-in-the-Cloud.

This manuscript describes the development of a resource module that is part of a learning platform named ``NIGMS Sandbox for Cloud-based Learning'' https://github.com/NIGMS/NIGMS-Sandbox. The overall genesis of the Sandbox is described in the editorial NIGMS Sandbox [1] at the beginning of this Supplement. This module delivers learning materials on the analysis of bulk and single-cell ATAC-seq data in an interactive format that uses appropriate cloud resources for data access and analyses.

## INTRODUCTION

Pathway analysis is a powerful computational approach used in bioinformatics and genomics research to gain insight into the underlying biological mechanisms involved in a particular biological process or disease. In comparison to differential expression analysis at the gene-level, which typically returns a lengthy list of differentially expressed (DE) genes across multiple conditions (e.g. normal versus cancer), pathway analysis offers a more systematic way to report and interpret these results. It allows researchers to annotate these DE genes into many certain functions and examine the interactions and functional relationships between genes, proteins and other molecular entities within biological pathways. By grouping the genes into subsets that share similar functions, pathway analysis has shown its importance in numerous downstream analyses, including disease subtype identification [2, 3], personalized treatment or medication approaches [4–8], survival analysis [9, 10], space biology [11, 12] and more.

Thus far, there are more than 100 pathway analysis methods that have been proposed, utilizing various statistical and computational approaches [13–15]. These methods can be divided into two different categories: ‘non-topology-based’ and ‘topology-based’ methods. The former treats pathways as simple, unordered collections of genes, whereas the latter takes into account the dependencies and interactions between genes within pathways. Furthermore, each of these categories can be divided into smaller subgroups based on their specific input requirements. In essence, some methods are designed to handle data from a single-omic level, while others have the capability to integrate and analyze multi-omics data. However, the wide range of pathway analysis methods presents a challenge for researchers in selecting the most suitable method(s) for their specific experimental purposes. These methods are written in different programming languages, tested and deployed in diverse environments, necessitating varying computational knowledge for their execution. Therefore, there is a need for a training module that can provide users with uniform guidance on how to utilize these methods effectively. Such a module would assist researchers in expanding their research capabilities, navigating the variety of pathway analysis methods and facilitating their selection and usage in a consistent manner.

Moreover, as sequencing costs continue to decrease, it is becoming common to assay genomic information from different cohorts of patients or samples across multiple species. As a result, the amount of genomic data generated exponentially increased over the past decades, leading to the establishment of multiple billion-dollar data repositories for biomedical research, such as the National Center for Biotechnology Information Gene Expression Omnibus (NCBI GEO) [16, 17], The Cancer Genome Atlas (GDC/TCGA) [18] and ArrayExpress [19, 20]. Leveraging these data, especially in the context of pathway analysis, requires expertise in data processing and substantial computational resources. Necessary resources include storage capacity for data, powerful Central Processing Units (CPUs) and/or Graphical Processing Units to efficiently analyze large-scale genomic data. However, these requirements often come at high costs, making them hard to afford for small research laboratories or educational purposes [21, 22]. To address this issue, cloud computing platforms have been adopted by many research communities to access computing and storage resources at a scale that had previously been available to only the largest research institutions, due to the well-known advantages of flexibility, elasticity and economy it can offer [23].

In this article, we present Cloud-based Consensus Pathway Analysis (CCPA), a specialized cloud-based self-learning module designed for students, researchers and clinicians, among others, to utilize cloud computing resources to perform pathway analysis and meta-analysis. The five core learning submodules of CCPA are as follows: (i) data acquisition and processing, which prepares and processes gene expression data, (ii) differential analysis and consensus analysis using four different methods, (iii) geneset and pathway acquisition, which guides users to effectively browse, explore and automatically download pathway information from Gene Ontology (GO) [24], Kyoto Encyclopedia of Genes and Genomes (KEGG) [25] and Reactome [26], (iv) pathway analysis, which performs pathway analysis using eight methods and (v) meta-analysis, which combines analysis results of related experiments and explores significantly impacted pathways across multiple analyses.

The learning module is designed to be self-contained and there is no prerequisite required for users to complete the module. Particularly, we provide detailed instructions on how to install and utilize the necessary software packages, as well as how to access and utilize the cloud resources. Through practical examples, code implementations and detailed explanations, users gain a comprehensive understanding and proficiency in utilizing current technological advancements in cloud computing via the Google Cloud for pathway analysis and other large-scale genomic analyses involving various biomedical species (e.g. human, mouse, fruit fly, zebrafish). Each code snippet provided in the learning module only takes, at most, some minutes to execute. The learning module is designed so that students can learn the materials without waiting too long for the analyses.

This article is a part of the special issue NIGMS SandBox Special Edition that presents NIGMS Sandbox (https://github.com/NIGMS/NIGMS-Sandbox), the collective effort aiming to encourage students and researchers to utilize cloud computing for the benefit of life science applications and research. Compared with our previously published web application CPA [27], there are numerous distinct differences in this cloud-based learning module CCPA. In addition to the structural differences (cloud-based training module versus web application), the topics covered in CCPA are wider (data processing, differential analysis, pathway database processing, pathway analysis, consensus pathway analysis). CCPA aims to expose students to the full analysis pipelines on the cloud with detailed instructions, source code and explanations for each step and parameter needed. These are necessary for learners to customize their analysis pipelines without relying on a single method or analysis tool.

The article is structured as follows: the *Methods* section outlines the cloud-based learning module’s overall design architecture and comprehensively covers data analyses incorporated in the module. The *Results* section provides a demonstration of CCPA, utilizing two Alzheimer’s disease datasets. The codes responsible for producing the results and figures discussed in this section can be found in the Supplementary Note. However, it is worth mentioning that this only presents a portion of CCPA, while the complete codes and explanations are available at https://github.com/NIGMS/Consensus-Pathway-Analysis-in-the-Cloud. Finally, in the *Discussion and Conclusion* section, we highlight our contributions to the research community, discuss some limitations of the current module and propose potential further enhancements to advance the module.

## METHODS

**Figure 1:**
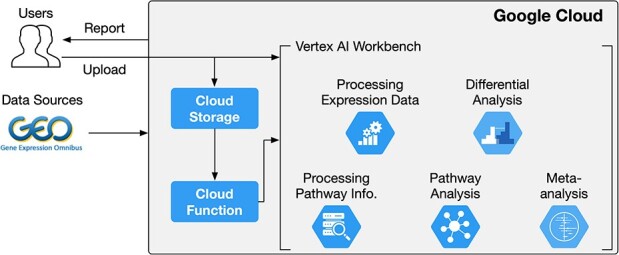
The high–level description of the CCPA pipeline. Users start by creating Google Cloud Storage buckets to store their data, preparing for the upcoming analysis. Next, they launch a Vertex AI workbench with R kernel, running Jupyter Notebooks that are cloned from our project repository. These Notebooks contain code and instructions for five separate analysis submodules. The Processing Expression Data submodule demonstrates data retrieval from public repositories, followed by preprocessing. The processed data are stored both locally in the Vertex AI workbench and in the user’s Google Cloud Storage Bucket. Subsequently, the Differential Analysis, Processing Pathway Information, Pathway Analysis and Meta-analysis submodules build upon the outputs of the Data processing submodule. Their results are also saved to the local repository in the Vertex AI workbench before being conveniently copied to the user’s cloud bucket. Users can further export analysis reports from their cloud buckets using various cloud functions.

Figure 1 shows the overall architecture of CCPA. The learning takes place within the Google Cloud environment via using a Vertex AI Workbench virtual machine with R kernel. Users have the flexibility to select the R version they prefer during the creation of the workbench. Upon creation, the working environment is automatically set up. The module consists of five submodules, presented as separate Jupyter Notebooks containing detailed code and instructions. Users can either upload these notebooks to the Vertex AI Workbench or clone them from the project’s GitHub repository before executing the source code directly within the notebooks.

In the first submodule, named Processing Expression Data, we provide guidance on acquiring data from NCBI GEO using both the web interface and R command line. For users with their own data or those preferring data from other repositories such as GDC/TCGA, we direct them to upload the required data files to the cloud environment via the web interface. Following data preparation, users are instructed on data processing, normalization and gene mapping. These processed data serve as input for subsequent submodules, including Differential Analysis, Processing Pathway Information, Pathway Analysis and Meta-analysis. Instructions are also given on saving all data, code and analysis results to the local repository in the Vertex AI Workbench as well as the user’s cloud storage bucket. The local repository on the Vertex AI virtual machine is accessible across all submodules, and code for transferring content between the storage bucket and the Vertex AI virtual machine is provided. This allows users to easily access the results of each submodule and copy them to their cloud bucket for further analysis or sharing with collaborators, as well as to pause and resume the analysis at any time.

### Cloud environment settings

Our learning module requires users to have a Google Cloud account. Google Cloud is a platform that provides a web-based, graphical user interface that we can use to access various computing resources and utilize them for many applications such as web servers or software deployment. In our learning module, we introduce the use of two Google Cloud services, namely Google Cloud Storage and Vertex AI Workbench.

#### Google cloud storage

**Figure 2:**
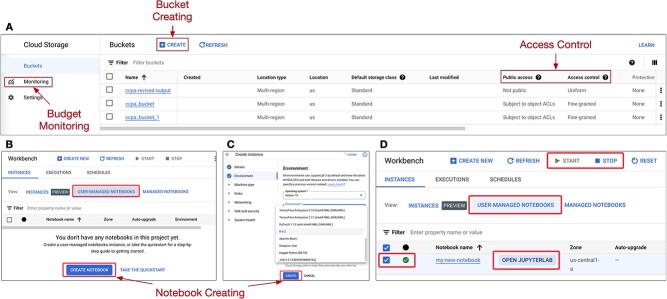
Setting up cloud environment. (A) Users start by creating Google Cloud Storage buckets to store their data and manage control access to share with their collaborators. (B) Users can create a new notebook on Google Cloud on the Workbench screen. (C) They can then configure the virtual machine with R version 4.2 as the development environment, with 4 CPUs and 15GB RAM. (D) When users finish creating a notebook, they can locate it by navigating to **USER–MANAGED NOTEBOOKS** tab. Click the **START** button on the top menu bar to start the machine. The starting process might take up to several minutes. When it is done, the green checkmark indicates that your virtual machine is running. Next, click **OPEN JUPYTER LAB** to access the notebook. Note that the Google Cloud interface may change over time, users are advised to refer to the Google Cloud documentation for the latest instructions in using Vertex AI Workbench.

Google Cloud Storage is a versatile object storage service and a cost-effective solution for data storage, which can be used in many applications such as static website hosting, data backup, content distribution and big data analytics. Figure 2A shows the screenshot of Google Cloud Storage’s user-friendly GUI. Through the GUI, we guide users on creating Google Cloud Storage Buckets for user data, public repositories and analysis-generated data. Users can easily upload their data to the buckets via this interface. We also stress data security, access control, storage classes and highlighted use cases such as web hosting, backup, content distribution and data analysis. This knowledge empowers users to effectively manage their data on Google Cloud.

#### Vertex AI workbench

Each learning submodule is organized as an R Jupyter Notebook, ensuring an interactive and practical experience for participants. The notebooks are executed within the Google Cloud environment, managed through Vertex AI Workbench, offering seamless accessibility without the need for extensive configurations. Learners can conveniently open the notebooks in their browser tabs, making it effortless to dive into the world of data analysis. We have provided details on configuring the virtual machine and working environment in our project repository. Users have the flexibility to create different machines that align with their data analysis needs.

Figure 2B and C show an example of what settings are available when creating a new notebook. For example, users can create the Jupyter Notebook (i.e. virtual machine) using R kernel version > 4.1 on a standard machine with a minimum configuration of 4 vCPUs, 15 GB RAM and 10GB of HDD, within the Workbench screen. Once the virtual machine is created, users have the option to start, stop or delete the notebook on the Workbench screen. The Workbench page also provides status information about the notebook, such as the current status (starting, running or stopped), and the time the notebook was created, as well as a link to access JupyterLab (Figure 2D) to start or resume the analysis. However, it is important to note that the Google Cloud interface may change over time; users are advised to refer to the Google Cloud documentation for the latest instructions on using Vertex AI Workbench.

#### Synchronizing data to google cloud bucket

In addition to using the web interface, we offer a command-line option for automation in transferring data between Google Cloud Storage and Vertex AI Instances. For example, to create a new bucket, users can use the following command in the R console:


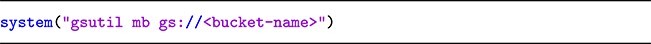


where **<bucket-name>** is the name of the bucket. To upload files to the bucket, we can use the following command:


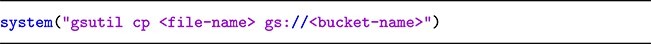


where **<file-name>** is the name of the file to be uploaded. Similarly, to download files from the bucket, we can use the following command:


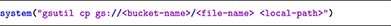


where **<local-path>** is the path to the local directory where the file will be downloaded. At any point in the analysis, users can save the snapshot of the R environment to the bucket using the following command: 


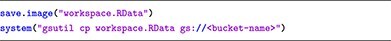


where **workspace.RData** is the name of the snapshot file. To load the snapshot from the bucket, we can use the following command:


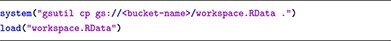


### Submodule 01: processing expression data

**Figure 3:**
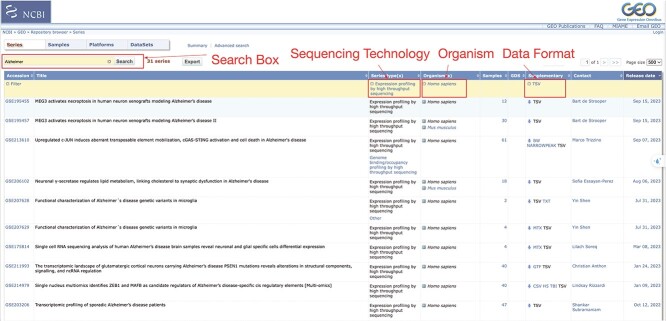
GEO dataset screening. Users can explore the GEO datasets using the database’s web interface. Within this interface, users can access metadata containing key information such as sequencing technology, organism, number of samples, data format and more. By interacting with these metadata columns, users can apply filters to narrow down datasets most suitable for their analysis needs.

The main goal of this submodule is to prepare the data for both differential analysis and gene set enrichment analysis. This involves the following steps: (i) browsing and downloading data from NCBI GEO using the web interface and R command line, (ii) uploading users-provided data to the Cloud, (iii) data processing and (iv) gene mapping. In the first step, we guide users in efficiently searching and exploring the NCBI GEO database to find relevant datasets using various criteria, such as keywords, conditions, tissues, organisms and platforms. Figure 3 shows the interface of the GEO database for searching the datasets. By using interactively filtering on the interface, users can easily uncover datasets that align with their specific research questions and inform decisions on which datasets to download and process. To facilitate dataset acquisition, we offer different methods for downloading data from NCBI GEO. Users can download data directly from the database’s website or utilize an R package, namely GEOquery [28], for downloading from the database.

However, it is important to note that we limit the description of NCBI GEO to the first step only. The purpose is for new users to get familiar with gene expression meta-data that have already been stored at NCBI GEO in the past decades. Thus, in the second step, we describe how users can upload their own data directly to Vertex AI Instance and Google Cloud Storage. In this step, we also clearly describe the format of the files to be uploaded, including the gene expression data and experiment design (sample grouping that describes the conditions, such as healthy versus disease). Users can certainly download data from GDC/TCGA or other public repositories, and upload the data in the format specified in the second step.

In the third step, we walk users through the process of handling provided datasets based on the repository, data type, platform and species. This involves actions such as delving into metadata and assessing data distribution. As a result, users can determine whether additional processing of their data is necessary. This step also covers common procedures for quality control and normalization. The process encompasses procedures such as quartile filtering to eliminate outliers and missing expression values, log normalization to ensure consistent sample distributions, and the utilization of **boxplot** function to verify the effectiveness of data normalization. Of note, the submodule is designed to handle table data generated from either microarray or RNA-Seq experiments. For those starting with raw sequencing files (.CEL for microarray or .FASTQ for RNA-Seq), we recommend users consult relevant protocols for alignment and obtaining the expression data table.

In the last step, we guide users in performing the gene ID mapping process. The reason is that pathway analysis requires both pathways (or gene sets) and expression data to be represented using the same type of gene IDs. However, it is often the case that the available data do not conform to this requirement. For example, while pathways from the KEGG database predominantly utilize Entrez Gene IDs, microarray datasets commonly employ probe set IDs or RefSeq transcript IDs. Consequently, we must perform a mapping process to convert the probe set IDs to Entrez Gene IDs. To accomplish this, we advise users to refer to the documentation of the platform used to generate the sequencing data, which provides information on the probe set IDs presented in the dataset. In some cases, data repositories such as NCBI GEO may include this documentation along with the expression data, making it readily accessible. Fortunately, this document already includes the essential mapping information from probe set IDs to other commonly used gene IDs, such as Entrez Gene IDs or gene Symbols. Alternately, we also guided users to download and utilize the annotation software packages, which are usually available on Bioconductor. For instance, AnnotationDbi [29] is a package that provides an interface for connecting and querying various annotation databases using SQLite data storage. hgu133plus2.db [30] is a package that is built upon a database to perform Probe IDs to gene Symbols mapping for human data.

### Submodule 02: differential analysis

The next step in pathway analysis involves conducting differential analysis, which aims to identify genes that exhibit significant expression changes between two or more biological conditions. The identification of these DE genes provides valuable insights into the biological processes influenced by the conditions of interest [31, 32].

This submodule introduces comprehensive instructions on utilizing well-established methods for differential analysis, tailored to different data types, including: t–test, limma [33], DESeq2 [34] and edgeR [35]. Furthermore, for microarray data, we recommend using the limma method, while for RNA-Seq data, we suggested utilizing DESeq2 and edgeR. These methods have proven their efficacy in detecting genes with noteworthy expression changes across various studies [36, 37].

Additionally, we emphasize the importance of visualizing the results of the differential expression analysis. Visualization techniques such as heatmaps and Volcano plots were introduced to assist users in conducting quality control checks, identifying potential outliers or noise in the data, and gaining valuable insights into the data distribution. These visualizations serve as valuable tools for pinpointing DE genes and evaluating the overall quality and reliability of the analysis.

### Submodule 03: processing pathway information

In this module, we cover three primary curated pathway databases: GO [38], KEGG [39] and Reactome [40]. GO offers a structured vocabulary to describe gene attributes across organisms, categorized into Molecular Function, Biological Process and Cellular Component. KEGG encompasses databases for genomes, pathways, diseases, drugs and chemical substances, serving bioinformatics research and education, genomics, metagenomics and drug development. Reactome provides pathway knowledge and tools for data visualization, integration and analysis covering diverse topics including classical intermediary metabolism, signaling, transcriptional regulation, apoptosis and disease.

This submodule describes database structures, ID types and how to retrieve pathway annotations. For effective browsing, we introduce specific software packages. The topGO package [41] retrieves GO terms from gene IDs obtained through DE analysis, utilizing the Gene2GO databases. To retrieve KEGG pathways and gene sets via the R console, users can employ the KEGGREST package [42], providing a client interface to the KEGGREST server. To access the Reactome databases, the package ReactomeContentService4R [43] provides useful functions for querying pathways data through the Reactome Content Service API.

In terms of maintainability, we advise users to regularly update the relevant R packages (topGO, KEGGREST and ReactomeContentService4R). This ensures that they have access to the latest pathway information for their analyses. Users can also save the pathways to avoid repeated downloads in the future, as each download only takes a couple of minutes. However, we recommend that users refresh the pathways regularly to ensure that their pathways are up-to-date.

### Submodule 04: pathway analysis

In this module, we provide users with comprehensive guidance on performing enrichment analysis using a list of DE genes and curated pathways from public databases. Enrichment analysis (EA) is a powerful technique that allows us to gain valuable biological insights from lists of significantly altered genes [44, 45]. By identifying biological pathways or ontology terms that are enriched in a gene list beyond what would be expected by chance, EA helps us understand the underlying mechanisms of diseases and the genes and proteins associated with specific diseases or drug targets.

**Figure 4:**
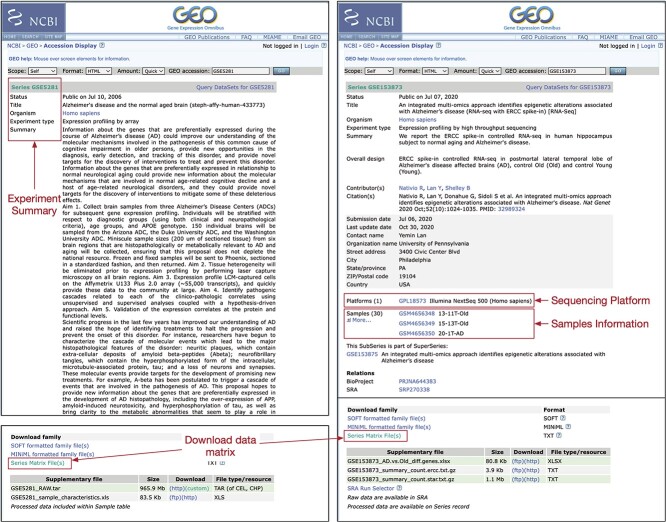
Querying data from GEO repository. On top of the web page showing the querying results, users can find details about the datasets, including their titles, publication dates, ownership and pertinent information related to the experimental design, such as organism, tissue, biopsy region, sample size and summaries. At the end of the web page, users can find further information about the dataset, such as the sequencing platform used, the number of samples, project ID and links to download the expression data. The left panel is the querying result for the microarray dataset (GSE5281), and the right panel is the querying result for the RNA-Seq dataset (GSE153873).

To provide users with a range of options, we introduce eight state-of-the-art enrichment analysis methods, including Wilcocon test [46], Kolmogorov–Smirnov (KS) test [47], Over-Representation Analysis (ORA/Webgestalt) [48, 49], Gene Set Analysis (GSA) [50], Pathway Analysis with Down-weighting of Overlapping Genes [51], Fast Gene Set Enrichment Analysis (FGSEA) [52], Correlation Adjusted MEan RAnk gene set test (CAMERA) [53] and Significance Analysis of Function and Expression (SAFE) [54]. To enhance the user experience and facilitate result interpretation, we also provide guidance on various visualization functions. These visualizations enable users to explore enriched gene sets and pathways in an intuitive and user-friendly manner, making the analysis process more accessible and informative.

### Submodule 05: meta-analysis

The decreased cost of high-throughput platforms has led to the generation of diverse datasets with varying sample sizes. This has created opportunities to overcome the limitations of small sample sizes through meta-analysis. Meta-analysis is a statistical analysis that systematically combines the results of multiple research studies. In the context of pathway analysis, meta-analysis enables the combination and analysis of multiple sample sets—even if they come from different platforms, as well as results obtained from multiple methods. By pooling analysis from various studies and from using multiple methods, meta-analysis enhances statistical power, increases sample size and strengthens the reliability and generalizability of research findings.

In this submodule, we guide users on performing generic inverse variance meta-analysis using **metagen** function available in the meta R package [55]. **metagen** is a statistical method used in meta-analysis to combine the results of multiple studies that have measured the same effect size, typically using different sample sizes and study designs. This method involves pooling the effect sizes from each study, weighted by the inverse of their variance, in order to obtain an overall estimate of the effect size and its confidence interval. Additionally, we provide instructions on producing plots, e.g. forest plots and pathway heatmaps, which allow users to compare enrichment scores obtained from meta-analysis using multiple datasets with those calculated from a single dataset. These visual tools aid in the assessment and comparison of the meta-analysis results, providing valuable insights into the combined data’s overall impact.

## RESULTS

In this section, we present a demonstration of CCPA using two Alzheimer’s disease datasets downloaded from NCBI GEO: GSE5281 [56] and GSE153873 [57], an Affymetrix microarray and RNA-Seq dataset, respectively. Figure 4 displays the GEO webpage with comprehensive dataset records, including crucial details such as titles, publication dates, ownership and experimental design info such as organism, tissue, biopsy region, sample size and summaries. Scrolling down, it presents more details such as sequencing platform, sample count, project ID and links to download expression data. In our example, GSE5281 used Affymetrix Human Genome U133 Plus 2.0 Array with 131 samples, and GSE153873 used Illumina NextSeq 500 with 30 samples. We can also click on sample IDs for detailed descriptions and clinical info of individual samples. The summary table at the bottom contains all experiment data. We can use the ‘(http)’ link to manually download raw data for all samples or choose ‘(custom)’ for specific samples of interest.

In the subsequent sections, we will demonstrate how to obtain these datasets and perform all the analyses using the R command line. Note that we will include some examples of code snippets and their results along with explanations such that users can better grasp the process and have a deeper understanding of the materials being taught in our learning module. We also provide codes to generate all the results and figures presented in the following sections in the Supplementary Note. This approach not only demonstrates what can be achieved from the module but also provides a hint about the hands-on learning experience users can expect. To run all the code provided, we used a standard machine with the configuration of 4 vCPUs, 15 GB RAM and 10GB of HDD created on Vertex AI Workbench.

### Submodule 01: processing expression data

To download the microarray dataset, GSE5281, from NCBI GEO, we utilize the **getGEO** function from the package GEOquery. This package is available in Bioconductor and can be installed through the following command:


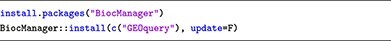


Once installed, we provide accession ID, i.e. GSE5281, to the **getGEO** function as its argument, as shown below


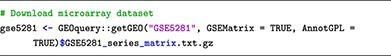


Alternatively, we have the option to employ the **fread** function from the data.table package to download data directly from its URL. The code block below demonstrates how we download the RNA-Seq dataset using this approach


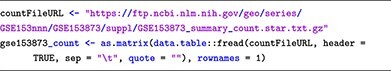


Subsequently, we conduct log normalization (base 2) and sample annotation (control versus disease) for downstream analyses. In our example, we perform data normalization only on the microarray dataset, and we use raw counts for the RNA-Seq dataset. Next, the Probe IDs are used as gene annotations for the microarray dataset, while the RNA-Seq dataset uses gene Symbols. The pathways in the following sections will use the gene Symbols. Therefore, we utilize the hgu133plus2.db package for mapping the gene IDs utilized in the datasets to gene Symbols used in pathway annotation. Finally, we obtain the necessary input data for the subsequent module’s analysis. These data include expression matrices, in which rows are gene Symbols and columns are samples, and sample grouping information specifying the condition of the samples.

### Submodule 02: differential analysis

This submodule includes the source code and instructions to perform differential analysis using t–test, limma, DESeq2 and edgeR. For each method, we provided a customized function such that it allows users to perform the analysis with one single command line. In each function, the parameters have default values that allow novice users to perform the analysis without setting those parameters. We have also added detailed explanations for each parameter used in each function so that users can read the instructions and change these parameters if they desire to do so. For example, we provided the following code snippet to run DESeq2:


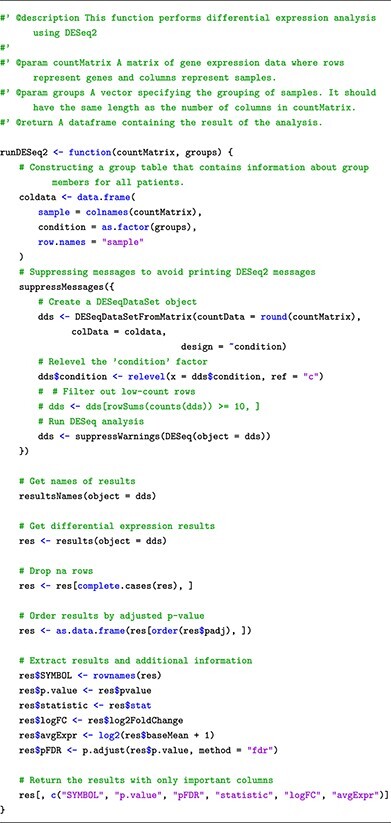


Accordingly, users only need to pass the expression matrix and sample grouping information obtained from Submodule 01 into the function arguments as **countMatrix** and **groups**, respectively. We have performed differential analysis using DESeq2 on the RNA-Seq dataset using the following code snippet:


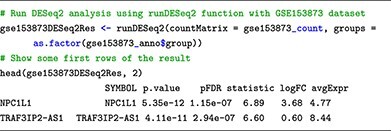


The codes for running differential analysis methods on the two datasets can be found in the Submodule 02 Jupyter Notebook and Supplementary Note. All methods return the tables of results that have genes represented as rows and their corresponding statistics, such as fold change, *P*-value and adjusted *P*-value for multiple comparisons, as columns. The columns have identical names across all methods. This will help users to generate different plots for visual representations of the findings by using our provided customized plot functions. These plots include: (i) MA plots to compare the average expression with the log2 fold-change, (ii) volcano plots to compare the log2 fold-change with −log10 *P*-value, (iii) Venn diagrams to compare DE genes from multiple analyses/datasets and (iv) gene heatmap to plot log2 fold-change and −log10 *P*-values across multiple analyses/datasets.

**Figure 5:**
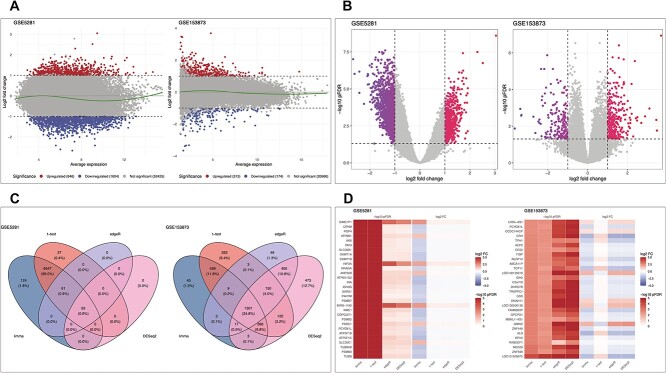
The visual representation of results obtained from the differential expression analysis. (A) The MA plot illustrates the relationship between gene average expression (*x*-axis) and fold change (*y*-axis). (B) The volcano plot showcases the relationship between gene fold change (*x*-axis) and statistical significance (*P*-value) (*y*-axis). For both the MA plot and the Vocalno plot, each point on the plot represents a gene, colored based on its significance. (C) The Venn diagram compares the list of DE genes identified by the four differential analysis methods (limma, t-test, edgeR and DESeq2) applied to the same dataset. (D) The heatmap displays the log2 fold-change of genes along with their −log10 *P*-values obtained from the four methods. In each plot, the microarray analysis result is shown on the left panel (GSE5281), while the RNA–seq analysis result is shown on the right panel (GSE153873).

Figure 5 displays the plots that users can obtain by following our learning submodule. The top panel of the figure (Figure 5A and B) shows the MA plot and volcano plot that visualizes the differential analysis results when applying limma on the microarray dataset (GSE5281) and DESeq2 on the RNA-Seq dataset (GSE153873). For the same dataset, users can use the four differential analysis techniques: limma, t-test, edgeR and DESeq2. To compare and contrast the analysis results, users can visualize all analyses using Venn diagrams or heatmaps (Figure 5D). Specifically, the Venn diagram (Figure 5C) can be used to compare the list of DE genes identified by the four differential analysis methods (limma, t-test, edgeR and DESeq2) applied on the same dataset. Similarly, the heatmap (Figure 5D) displays the log2 fold-change of genes along with their −log10 *P*-values obtained from the four methods.

### Submodule 03: processing pathway information

In this example, we curate pathway information from two public databases: GO, KEGG and Reactome. To achieve this, we use R packages named topGO, KEGGREST and ReactomeContentService4R. Additionally, for genome-wide annotation specific to human, we rely on the org.Hs.eg.db package.

To acquire GO terms, we explore the GO database through the topGO and org.Hs.eg.db package as follows:


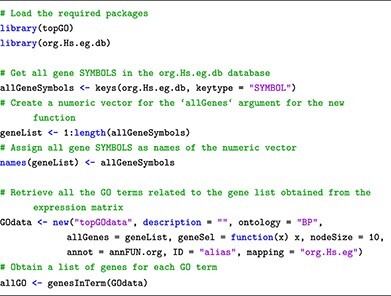


For obtaining KEGG pathways related to human, the **keggList** function from the KEGGREST package is employed. The respective command lines for these processes are as follows:


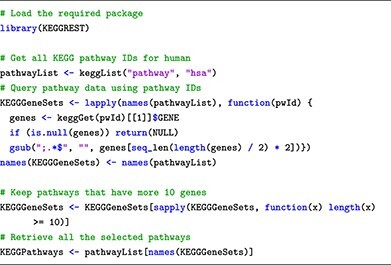


To retrieve human pathways from Reactome, we apply the function **getSchemaClass()** from ReactomeContentService4R package as follows:


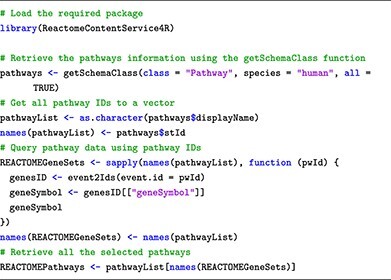


Lastly, the curated pathways are saved in the ‘.gmt’ format, which is a widely used file format compatible with various pathway analysis algorithms, enabling seamless integration into subsequent analyses.


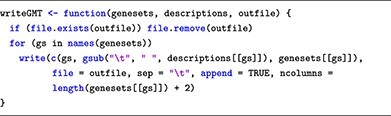


### Submodule 04: pathway analysis

For pathway analysis, we include in this learning submodule a total of eight enrichment methods: Wilcocon test [46], KS test [47], ORA/Webgestalt [48, 49], GSA [50], PADOG [51], FGSEA [52], CAMERA [53] and SAFE [54]. Similar to the functions for differential analysis and visualization introduced thus far, we also provide customized functions, with detailed explanations, that wrap the process of performing each enrichment method. Users can simply provide the differential analysis result obtained from Submodule 02 to run pathway analysis. For example, users can use the following function to perform FGSEA:


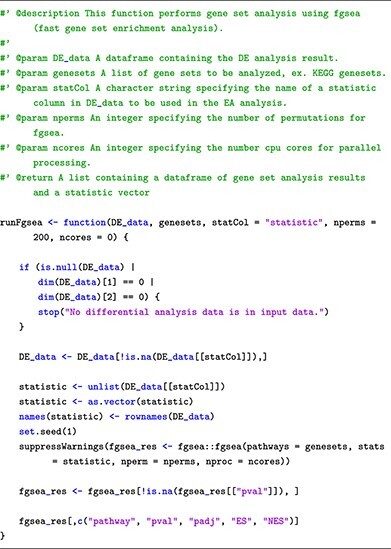


Users can find the codes and explanations for the remaining seven methods in our Submodule 04’s Jupyter Notebook. In our example, we perform pathway analysis using FGSEA on the two datasets by running the following code snippets:


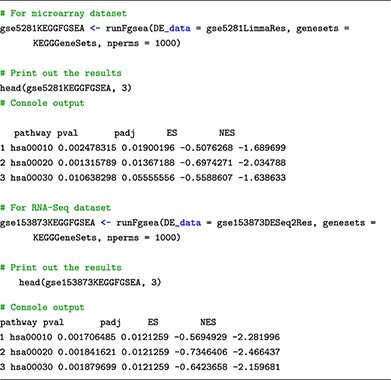


Finally, all functions introduced in this submodule return analysis results that are stored in a table format, with pathways represented as rows and their corresponding statistics, including *P*-value (pval), adjusted *P*-value (padj), enrichment score (ES) and normalized enrichment score (NES), as columns. The columns’ names are identical across all methods, which makes it easier to compare and integrate the results from multiple analyses, as outlined in the following submodule.

### Submodule 05: meta-analysis

In this submodule, we perform generic inverse variance meta-analysis using **metagen** function available in the meta package. The function allows us to effectively combine the enrichment scores of each pathway, which were obtained from the individual analyses. This enables us to gain a comprehensive understanding of pathway enrichment across both datasets and identifies any potential commonalities or differences in the biological processes at play. In the following code snippets, we showcase the meta-analysis of the results from using FGSEA on the two datasets.


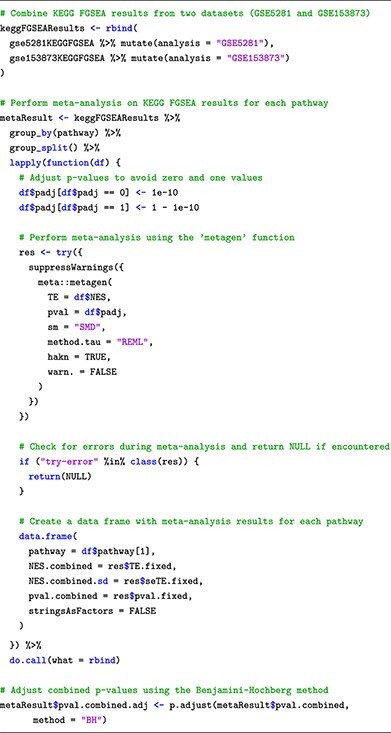


Similar to the Pathway Analysis submodule, the analysis results are also presented in a tabular format, where pathways are depicted as rows and their corresponding statistics, such as combined enrichment scores and associated *P*-values (adjusted for multiple comparisons), are represented as columns.


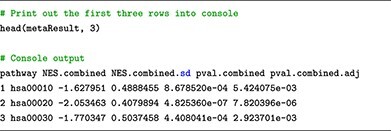


**Figure 6:**
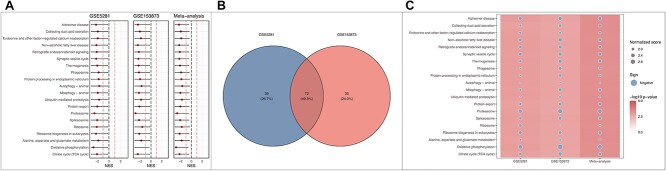
Visual representation for the results derived from applying FGSEA on the GSE5281 and GSE153873 datasets, along with their meta-analysis. (A) The forest plot displays the individual pathway analysis outcomes and their meta-analysis. Each pathway’s normalized score for each dataset is denoted by a circle, with horizontal segments depicting the confidence intervals around these scores. (B) Venn diagram compares significant pathways obtained from the two individual analyses. (C) Pathway heatmap to visualize enrichment scores and −log10 *P*-values obtained from the three analyses. The plots allow for a comprehensive visual comparison of pathway enrichment results across the datasets, facilitating insights into shared and distinctive pathways between the studies.

Finally, we also provided customized functions to visualize the individual analysis results and their meta-analysis result. Figure 6 showcases the plots obtained from running the plot functions provided in our learning module. The functions can be used to plot: (i) forest plot to display pathway’s ES with its confidence interval, (ii) Venn diagram to compare significant pathways obtained from multiple analyses/datasets and (iii) pathway heatmap to visualize enrichment scores and −log10 *P*-values obtained from one or multiple analyses/datasets. By employing the meta-analysis approach, we gain a more robust and comprehensive view of the pathway enrichment results, enabling us to draw more insightful conclusions by comparing the outcomes of the two datasets. The codes to reproduce the results and plots presented here can be found in the Supplementary Note.

### Storing data files in google cloud bucket

To store the data files in Google Cloud Bucket, we use the **gsutil** cloud application with the following syntax:


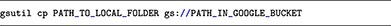


To execute this command line, we can either open the terminal or use the **system** function in R within a Jupyter Notebook’s code cell. For example, we use the following R command lines to save our analysis results in Google Cloud Bucket:


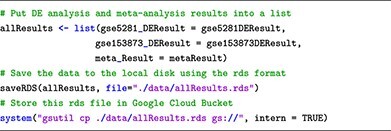


## DISCUSSION AND CONCLUSION

Cloud computing has become an essential tool for researchers to perform large-scale data analyses. However, the learning curve for cloud computing can be steep for researchers who are unfamiliar with the field of high-performance computing. With the increasing popularity of cloud computing, there is a need for training modules that can help researchers learn how to use cloud computing for their research. This is especially helpful with small research groups that do not have consistent access to high-performance computing resources. The biggest advantage of cloud computing is that it provides a cost-effective solution for the necessary computational resources with the ability to scale up or down as needed without the need to purchase and maintain expensive hardware. It also allows researchers to perform their analysis in a reproducible manner since the computing environment can be easily replicated. Another big advantage of cloud computing is that it allows researchers to share their analysis with other researchers with ease, which is especially important for researchers who are working on collaborative projects.

The learning module introduced in this article has been derived from the web-based application for pathway analysis, known as CPA, previously developed by the authors. Genomic datasets are often massive, and pathway analysis requires significant computational resources. Cloud computing provides the necessary infrastructure to perform these analyses that bypass the limitations of local computational resources. Therefore, by extending core analysis modules of our CPA on Google Cloud, we have provided a complete cloud-based training module on pathway analysis, which allows researchers to uncover meaningful biological insights from their data in a timely and efficient manner. This learning module also serves as an example of using cloud computing for collaborative research between computer scientists and biologists across different institutions and agencies. By leveraging the cloud computing infrastructure, users can effectively reduce the time and effort required to set up the computing environment before they can start learning. For example, one may need to download software and dependent packages, and prepare the computing environment before performing any analysis. Many of those steps can be time-consuming, especially for novice learners and students with life-science backgrounds. When using cloud infrastructure for learning, teachers or organizers can pre-specify a virtual machine with all configurations needed (software, hardware, dependency, computing power). Users only need to use the predefined and well-tested specifications of virtual machines and computing environments to perform the analyses described in the learning module.

In this learning module, we have included here five submodules that cover different aspects of pathway analysis. The submodules are designed to be taken in order, as each submodule builds on the previous one. The first submodule, Processing Expression Data, teaches the fundamental concepts of preprocessing and quality control of data obtained from microarray or RNA-Seq experiments. As genomic datasets can come from different platforms with various formats and normalization methods, it is important to learn how to process and normalize the data before performing any downstream analysis. The second submodule, Differential Analysis, focuses on how to analyze gene expression data to identify genes that are DE between two or more conditions using multiple techniques. The module was designed to handle different types of data, including microarray and RNA-Seq data, and different normalization methods. The third submodule, Processing Pathway Information, shows us how to use different R libraries to automatically obtain pathways and GO terms with associated gene lists. The fourth submodule, Pathway Analysis, focuses on the identification of enriched biological pathways or functional categories within a set of genes of interest. This allows researchers to familiarize themselves with the different tools and choose the most appropriate one for their data. Finally, the fifth submodule, meta-analysis, teaches the techniques of meta-analysis, which is a statistical approach to combine the results of multiple independent studies. Reaching a consensus on the results of multiple studies can be challenging, and meta-analysis provides a solution to this problem by combining the results of multiple studies to obtain a more accurate and robust result. Moreover, each code snippet provided in the submodule’s Jupyter Notebook only takes some seconds or, at most, some minutes to execute. The methods chosen for differential analysis and pathway analysis are relatively fast. Therefore, the learning module is also designed so that students can learn the materials without waiting too long for the analyses.

Overall, the cloud learning module provides a comprehensive and practical guide for researchers to systematically analyze and interpret their genomic data using various bioinformatics tools and techniques. Users can also learn how to leverage the large storage on the cloud to save their data and analysis results. However, it is important to note that the learning materials could be overwhelming for individuals who are new to bioinformatics and cloud computing. Despite the user-friendly approach, some researchers might still find certain concepts or tools daunting, which might impede their journey toward proficiency. In addition, while the convenience of sharing data and analysis in the cloud is a great advantage, it also poses a risk to the security of personal data if the users do not set up their accounts properly. To ensure the security of personal data, each user should use their personal account to set up virtual machines and storage buckets that are not visible to any other users. When sharing the data with other users, we recommend that the users make fine-grained access control to their data following the guidelines from the Google Cloud Platform. As part of our future endeavors, we aim to transition our web-based CPA’s computing engine to cloud-based infrastructure. This will enable users to conveniently access CCPA through a user-friendly GUI/web interface, allowing them to effortlessly perform consensus pathway analysis with a single click.

**Key Points**

- Cloud-based computing provides cost-effective computational resources for analyzing large-scale genomic data.
- This article presents five cloud-based submodules that enable users to learn and perform consensus pathway analysis.
- This article assists life scientists in performing differential analysis and pathway analysis.
- This article allows users to integrate analysis results obtained from multiple analyses to find consensus results.

## Supplementary Material

Supplementary_Note_bbae222

## Data Availability

The data used in this pipeline can be found at NCBI GEO database website with accession IDs: GSE5281 and GSE153873.

## Notes

**Ha Nguyen** is a PhD student in the Department of Computer Science and Software Engineering at Auburn University, Auburn, Alabama, USA. His research foci include single-cell data deconvolution and systems-level analysis.

**Van-Dung Pham** is a PhD student in the Department of Computer Science and Software Engineering at Auburn University, Auburn, Alabama, USA. His research interests include cancer subtyping and systems-level analysis.

**Hung Nguyen** is a PhD student in the Department of Computer Science and Software Engineering at Auburn University, Auburn, Alabama, USA. His research interests include cancer subtyping, network inference and single-cell data analysis.

**Bang Tran** is an Assistant Professor in the Department of Computer Science at California State University, Sacramento, California, USA. His research foci include single-cell data deconvolution and systems-level analysis.

**Juli Petereit** is a Bioinformatics and Data Scientists. She is the director of the Nevada Bioinformatics Center and Nevada INBRE Data Science Core at the University of Nevada, Reno, USA.

**Tin Nguyen** is an Associate Professor in the Department of Computer Science and Software Engineering at Auburn University, Auburn, Alabama, USA. His research focuses on developing machine learning and statistical methods that can be applied to bioinformatics and computational biology.
