# Supplementary material for: CCPA: cloud-based, self-learning modules for consensus pathway analysis using GO, KEGG and Reactome
Source: Brief Bioinform. 2024 Jul 23;25(Suppl 1):bbae222. doi: 10.1093/bib/bbae222 (PMC11264295; doi:10.1093/bib/bbae222)

CCPA: Cloud-based, self-learning modules for Consensus Pathway Analysis using GO, KEGG, and REACTOME Supplementary Note

Ha Nguyen,[1] Van-Dung Pham,[1] Hung Nguyen,[1] Bang Tran,[2] Juli Petereit,[3] and Tin Nguyen[1,*]

[1]Department of Computer Science and Software Engineering, Auburn University, Auburn, AL,
[2]Department of Computer Science, California State University, Sacramento, CA,
[3]Nevada Bioinformatics Center, University of Nevada, Reno, NV,
[*]Corresponding author: tinn@auburn.edu

March 15, 2024

Contents

1 Submodule 01: Processing Expression Data . . . 3
- 1.1 Download and process Microarray dataset GSE5281 . . . 3
- 1.2 Download and process RNA-Seq dataset GSE153873 . . . 6

2 Submodule 02: Differential Analysis . . . 6
- 2.1 Differential Analysis using limma . . . 6
  - 2.1.1 Microarray Dataset: GSE5281 . . . 8
  - 2.1.2 RNA-Seq Dataset: GSE153873 . . . 10
- 2.2 Differential Analysis using t-test . . . 12
  - 2.2.1 Microarray Dataset: GSE5281 . . . 13
  - 2.2.2 RNA-Seq Dataset: GSE153873 . . . 13
- 2.3 Differential Analysis using edgeR . . . 14
  - 2.3.1 Microarray Dataset: GSE5281 . . . 15
  - 2.3.2 RNA-Seq Dataset: GSE153873 . . . 15
- 2.4 Differential Analysis using DESeq2 . . . 15
  - 2.4.1 Microarray Dataset: GSE528 . . . 17
  - 2.4.2 RNA-Seq Dataset: GSE153873 . . . 17
- 2.5 Differential Analysis Result Visualization . . . 18
  - 2.5.1 MA Plot . . . 18
  - 2.5.2 Volcano Plot . . . 21
- 2.6 Gene-level Consensus Analysis . . . 24
  - 2.6.1 Gene Venn Diagram . . . 25
  - 2.6.2 Gene Heatmap Plot . . . 27

3 Submodule 03: Processing Pathway Information . . . 30
- 3.1 Retrieving GO Terms . . . 31

3.2 Retrieving Pathways from KEGG Database 34
3.3 Retrieving Pathways from REACTOME Database 35

4 Submodule 04: Pathway Analysis **39**
4.1 Running FGSEA on Microarray Dataset: GSE5281 40
4.2 Running FGSEA on RNA-Seq Dataset: GSE153873 41

5 Submodule 05: Meta-analysis **41**
5.1 Perform Meta-analysis 41
5.2 Visualization of Analysis Result 43
5.2.1 Forest Plot 43
5.2.2 Pathway Venn Diagram 45
5.2.3 Pathway Heatmap Plot 47

1 Submodule 01: Processing Expression Data

1.1 Download and process Microarray dataset GSE5281

```
# Install and import packages for data downloading
suppressMessages({

    if (!require("BiocManager", quietly = TRUE)) {
        suppressWarnings(install.packages("BiocManager"))

    }
    suppressWarnings(BiocManager::install("GEOquery", update = F))
    suppressWarnings(BiocManager::install("Biobase", update = F))

})
suppressMessages(library("GEOquery"))
suppressMessages(library("Biobase"))

# Downloading GEO microarray data using dataset accession ID
accession_ID <- "GSE5281"
suppressMessages({gse5281 <- getGEO(GEO = accession_ID, GSEMatrix = TRUE,␣
↪AnnotGPL = TRUE)[[1]]})

# Extract the gene expression data and perform data normalization
gse5281_expr <- log2(Biobase::exprs(gse5281) + 1)
head(gse5281_expr, c(6, 6))
```

A matrix: 6 × 6 of type dbl

	GSM119615	GSM119616	GSM119617	GSM119618	GSM119619	GSM119620
1007_s_at	9.440814	9.658471	10.751230	10.704818	11.124313	9.953529
1053_at	3.426978	4.325694	3.813565	3.968298	7.125949	3.033289
117_at	3.842662	4.264332	6.620960	6.632238	6.747769	6.639674
121_at	7.443710	8.181477	9.071952	8.958108	8.903476	8.476106
1255_g_at	5.744617	6.799294	6.644170	4.194306	9.298787	6.731381
1294_at	6.103364	6.611478	6.272061	7.576066	8.116222	7.034395

```
# Extract necessary data for Gene Differential Analysis
gse5281_anno <- data.frame(
  sample = colnames(gse5281_expr),
  group = ifelse(grepl("normal", Biobase::pData(gse5281)$characteristics_ch1.8),␣
↪"c", "d"),
  region = make.names(Biobase::pData(gse5281)$characteristics_ch1.4)
)
head(gse5281_anno)
```

A data.frame: 6 × 3

	sample <chr>	group <chr>	region <chr>
1	GSM119615	c	Organ.Region..Entorhinal.Cortex.
2	GSM119616	c	Organ.Region..Entorhinal.Cortex.
3	GSM119617	c	Organ.Region..Entorhinal.Cortex.
4	GSM119618	c	Organ.Region..Entorhinal.Cortex.
5	GSM119619	c	Organ.Region..Entorhinal.Cortex.
6	GSM119620	c	Organ.Region..Entorhinal.Cortex.

```r
# Install the genome wide annotation database for human
suppressMessages({
    suppressWarnings({
        if (!require("BiocManager", quietly = TRUE))
            install.packages("BiocManager")
            BiocManager::install("hgu133plus2.db", update = F)
    })
})
# Import hgu133plus2.db package
library(hgu133plus2.db)

#' @description This function maps identifiers in a dataframe using a mapping␣
↪dataframe.
#'
#' @param data_df The dataframe containing the data to be mapped.
#' @param mapping_df The dataframe containing the mapping information.
#' @param data_source_col The column name in data_df containing the identifiers␣
↪to be mapped (default: "PROBEID").
#' @param data_target_col The column name to use for the mapped results in the␣
↪output dataframe. If NULL, it uses the same name as data_source_col.
#' @param data_result_col The optional column name to use for the mapped results␣
↪in the output dataframe. If provided, it will replace data_target_col.
#' @return A dataframe with mapped identifiers.
#'
# Function to map identifiers, such as probe IDs, to gene symbols using a␣
↪mapping dataframe.
map_identifiers <- function(data_df, mapping_df, data_source_col = "PROBEID",␣
↪data_target_col = "SYMBOL", data_result_col = NULL) {

    # Merge data_df with mapping_df based on data_source_col
    data_df = merge(mapping_df, data_df, by = data_source_col)
    # Remove rows with NA values in the data_target_col
    data_df <- data_df[!is.na(data_df[, data_target_col]), ]
    # Remove duplicated gene symbols, keeping the first occurrence
    data_df <- data_df[!duplicated(data_df[[data_target_col]], fromLast =␣
↪FALSE), ]
    # Set row names to the values in data_target_col
    rownames(data_df) <- data_df[[data_target_col]]
```

```r
    # Drop columns from mapping_df that are merged into the result dataframe
    if (!is.null(data_result_col)) {
        data_df[[data_result_col]] <- data_df[[data_target_col]]

        # Check if data_result_col is the same as data_target_col
        if (data_result_col == data_target_col) {
            data_df <- data_df[, !(names(data_df) %in% colnames(mapping_df)[1:
↪2])]
        } else {
            data_df <- data_df[, !(names(data_df) %in% colnames(mapping_df))]
        }
    }

    return(data_df)
}

## Gene ID mapping for GSE5281 dataset
GSE5281Genes <- rownames(gse5281_expr)
GSE5281GenesMapping <- suppressMessages(AnnotationDbi::select(x = hgu133plus2.
↪db,
                                        keys = GSE5281Genes,
                                        columns = c("PROBEID", "SYMBOL")))
colnames(GSE5281GenesMapping) <- c("FROM", "SYMBOL")
# Convert the gene expression to data.frame
gse5281_expr <- as.data.frame(gse5281_expr)
# Create a column to contain the gene id (name should match the column in the␣
↪mapping table)
gse5281_expr$FROM <- rownames(gse5281_expr)
# Use the map_identifiers to map the current gene id to the target gene id
gse5281_expr <- map_identifiers(data_df = gse5281_expr, mapping_df =␣
↪GSE5281GenesMapping,
                    data_source_col = "FROM", data_target_col = "SYMBOL",␣
↪data_result_col = "SYMBOL")
head(gse5281_expr, c(6, 6))
```

A data.frame: 6 × 6

	GSM119615 <dbl>	GSM119616 <dbl>	GSM119617 <dbl>	GSM119618 <dbl>	GSM119619 <dbl>	GSM119620 <dbl>
DDR1	9.440814	9.658471	10.751230	10.704818	11.124313	9.953529
RFC2	3.426978	4.325694	3.813565	3.968298	7.125949	3.033289
HSPA6	3.842662	4.264332	6.620960	6.632238	6.747769	6.639674
PAX8	7.443710	8.181477	9.071952	8.958108	8.903476	8.476106
GUCA1A	5.744617	6.799294	6.644170	4.194306	9.298787	6.731381
UBA7	6.103364	6.611478	6.272061	7.576066	8.116222	7.034395

1.2 Download and process RNA-Seq dataset GSE153873

```
# Download the RNA-seq data
countFileURL <- "https://ftp.ncbi.nlm.nih.gov/geo/series/GSE153nnn/GSE153873/
↪suppl/GSE153873_summary_count.star.txt.gz"
gse153873_count <- as.matrix(data.table::fread(countFileURL, header = TRUE, sep␣
↪= "\t", quote = ""), rownames = 1)
# Normalize count data for limma and t-test in the differential analysos
gse153873_count_normalized <- log2(gse153873_count +1)
head(gse153873_count, c(6, 6))
```

A matrix: 6 × 6 of type int

	20-1T-AD	13-11T-Old	15-13T-Old	16-14T-Old	3-17T-Young	5-18T-Young
SGIP1	1405	1405	1169	2408	859	1164
NECAP2	295	460	334	347	617	585
AZIN2	356	306	385	507	787	751
AGBL4	191	200	173	323	36	89
CLIC4	876	1443	639	792	4806	5968
SLC45A1	291	329	298	636	139	204

```
# Extract necessary data for Gene Differential Analysis
gse153873_anno <- data.frame(
  sample = colnames(gse153873_count),
  group = ifelse(grepl("AD", colnames(gse153873_count)), "d", "c")
)
head(gse153873_anno)
```

A data.frame: 6 × 2

	sample <chr>	group <chr>
1	20-1T-AD	d
2	13-11T-Old	c
3	15-13T-Old	c
4	16-14T-Old	c
5	3-17T-Young	c
6	5-18T-Young	c

2 Submodule 02: Differential Analysis

2.1 Differential Analysis using limma

```
# Install limma package
suppressMessages({
    if (!require("BiocManager", quietly = TRUE)) {
        suppressWarnings(install.packages("BiocManager"))
    }
    suppressWarnings(BiocManager::install("limma", update = T))
```

```r
})
# Import limma package
suppressPackageStartupMessages({
    library("limma")
})
```

```r
#' @description This function performs differential expression analysis using␣
↪limma
#'
#' @param normExprs A matrix or data frame containing gene expression values.␣
↪Rows represent genes, and columns represent samples.
#' @param design A matrix or data frame specifying the experimental design for␣
↪the linear model. Each column corresponds to a different experimental factor␣
↪or covariate.
#' @param contrast A matrix or data frame defining the contrasts of interest for␣
↪the differential expression analysis. Each row represents a contrast.
#' @return A list containing a limma-fitted model and an analysis result␣
↪dataframe.

runLimma <- function(normExprs, design, contrast) {
    # if (max(normExprs) > 100) normExprs <- log2(normExprs + 1)

    # Fit a linear model
    fit <- lmFit(object = normExprs, design = design)
    # Specify contrasts for differential expression
    fit <- contrasts.fit(fit = fit, contrasts = contrast)
    # Empirical Bayes moderation of standard errors
    fit <- eBayes(fit)

    # Extract results and additional information
    DERes <- topTable(fit, coef = 1, number = nrow(normExprs), confint=TRUE)

    DERes$SYMBOL <- rownames(DERes)
    DERes$p.value <- DERes$P.Value
    DERes$statistic <- DERes$t
    DERes$avgExpr <- DERes$AveExpr
    DERes$pFDR <- DERes$adj.P.Val

    # Return a dataframe containing limma results
    limma_res = DERes[, c("SYMBOL", "p.value", "pFDR", "statistic", "logFC",␣
↪"avgExpr")]

}
```

2.1.1 Microarray Dataset: GSE5281

```
# Get the group information and convert it to factor
gse5281_anno$group <- factor(gse5281_anno$group)
# Create design matrix
design <- model.matrix(object = ~0 + group, gse5281_anno)
# Show the design matrix
design
```

A matrix: 161 × 2 of type dbl

	groupc	groupd
1	1	0
2	1	0
3	1	0
4	1	0
5	1	0
6	1	0
7	1	0
8	1	0
9	1	0
10	1	0
11	1	0
12	1	0
13	1	0
14	1	0
15	1	0
16	1	0
17	1	0
18	1	0
19	1	0
20	1	0
21	1	0
22	1	0
23	1	0
24	1	0
25	1	0
26	1	0
27	1	0
28	1	0
29	1	0
30	1	0
.	.	.
132	0	1
133	0	1
134	0	1
135	0	1
136	0	1
137	0	1
138	0	1
139	0	1
140	0	1
141	0	1
142	0	1
143	0	1
144	0	1
145	0	1
146	0	1
147	0	1
148	0	1
149	0	1
150	0	1
151	0	1
152	0	1

```
# Set up contrasts of interest and recalculate model coefficients
cont.matrix <- limma::makeContrasts("groupd-groupc", levels = design)
cont.matrix
```

A matrix: 2 × 1 of type dbl

	groupd-groupc
groupc	-1
groupd	1

```
# Run limma analysis using runLimma function
gse5281LimmaRes <- runLimma(normExprs = gse5281_expr, design = design, contrast␣
↪= cont.matrix)
# Show some first rows of the result tables
head(gse5281LimmaRes)
```

A data.frame: 6 × 6

	SYMBOL <chr>	p.value <dbl>	pFDR <dbl>	statistic <dbl>	logFC <dbl>	avgExpr <dbl>
TUBB	TUBB	1.432179e-22	3.177289e-18	-11.36377	-1.631276	9.123189
PSMB3	PSMB3	2.388997e-21	1.829653e-17	-10.92825	-1.408793	8.736749
TUBB4B	TUBB4B	2.474176e-21	1.829653e-17	-10.92281	-1.726316	10.297977
SLC35E1	SLC35E1	7.401763e-21	4.105203e-17	10.75253	1.627450	11.470567
ATP5F1C	ATP5F1C	9.950015e-21	4.414822e-17	-10.70648	-1.881757	10.012823
ATP5F1B	ATP5F1B	2.802296e-20	1.036149e-16	-10.54504	-1.616196	10.576243

2.1.2 RNA-Seq Dataset: GSE153873

```
# Get the group information and convert it to factor
gse153873_anno$group <- factor(gse153873_anno$group)
# Create design matrix
design <- model.matrix(object = ~0 + group, gse153873_anno)
# Show the design matrix
design
```

A matrix: 30 × 2 of type dbl

	groupc	groupd
1	0	1
2	1	0
3	1	0
4	1	0
5	1	0
6	1	0
7	1	0
8	0	1
9	0	1
10	0	1
11	0	1
12	0	1
13	1	0
14	1	0
15	1	0
16	1	0
17	1	0
18	1	0
19	1	0
20	1	0
21	1	0
22	1	0
23	0	1
24	1	0
25	0	1
26	0	1
27	0	1
28	0	1
29	0	1
30	1	0

```
# Set up contrasts of interest and recalculate model coefficients
cont.matrix <- limma::makeContrasts("groupd-groupc", levels = design)
# Show the contrast matrix
cont.matrix
```

A matrix: 2×1 of type dbl

	groupd-groupc
groupc	-1
groupd	1

```
# Run limma analysis using runLimma function
gse153873LimmaRes <- suppressWarnings(runLimma(normExprs =
 ↪gse153873_count_normalized, design = design, contrast = cont.matrix))
# Show some first rows of the result tables
head(gse153873LimmaRes)
```

A data.frame: 6 × 6

	SYMBOL <chr>	p.value <dbl>	pFDR <dbl>	statistic <dbl>	logFC <dbl>	avgExpr <dbl>
LOC101926975	LOC101926975	3.656235e-09	9.921193e-05	-8.344000	-1.8227116	3.023096
ZNF596	ZNF596	1.039453e-07	9.436325e-04	-7.031987	-0.9139531	6.144073
MED29	MED29	1.043264e-07	9.436325e-04	-7.030596	-0.7998715	10.203245
RABGEF1	RABGEF1	2.890031e-07	1.751271e-03	-6.646324	-0.3878036	9.562289
DPH2	DPH2	3.573454e-07	1.751271e-03	-6.566992	-0.7689566	7.747132
ALB	ALB	3.872351e-07	1.751271e-03	-6.537030	-2.0374944	3.125139

2.2 Differential Analysis using t-test

```
# Install matrixTests package
suppressMessages({
    suppressWarnings(install.packages("matrixTests", quiet = T))
})
# Import matrixTests package
suppressPackageStartupMessages({library("matrixTests")})
```

```
#' @description This function performs differential expression analysis using␣
↪t-test
#'
#' @param countMatrix A matrix of gene expression data where rows represent␣
↪genes and columns represent samples.
#' @param groups A vector specifying the grouping of samples. Should have the␣
↪same length as the number of columns in countMatrix.
#' @param alternative The type of alternative hypothesis for the t-test ("two.
↪sided", "less", or "greater").
#' @return A dataframe containing the result of the analysis.

runTtest <- function(countMatrix, groups, alternative = "two.sided") {
    # if (max(countMatrix) > 100) countMatrix <- log2(countMatrix + 1)

    grUnique <- unique(groups)
    # Divide the countMatrix matrix into two separate matrices X and Y. X is the␣
↪control group and Y is the disease group
    X <- countMatrix[, groups == grUnique[1]]
    Y <- countMatrix[, groups == grUnique[2]]

    # Perform Welch's t-test using row_t_welch function
    tTest_res <- suppressWarnings(row_t_welch(x = X, y = Y, alternative =␣
↪alternative, conf.level = 0.95))

    # Extract results and additional information
    tTest_res$SYMBOL <- rownames(tTest_res)
    tTest_res$p.value <- tTest_res$pvalue
```

```
    tTest_res$statistic <- tTest_res$statistic
    tTest_res$logFC <- log2(tTest_res$mean.y) - log2(tTest_res$mean.x)
    tTest_res$avgExpr <- rowMeans(countMatrix)

    # Adjust p-values for multiple testing using the false discovery rate (FDR)
    tTest_res$pFDR <- p.adjust(tTest_res$p.value, method = "fdr")

    # Return the results with only important columns
    tTest_res[, c("SYMBOL", "p.value", "pFDR", "statistic", "logFC", "avgExpr")]
}
```

2.2.1 Microarray Dataset: GSE5281

```
# Run t-test analysis using runTtest function with GSE5281 dataset
gse5281TtestRes <- runTtest(countMatrix = gse5281_expr, groups = as.
↪factor(gse5281_anno$group))
# Show some first rows of the result tables
head(gse5281TtestRes)
```

A data.frame: 6 × 6

	SYMBOL <chr>	p.value <dbl>	pFDR <dbl>	statistic <dbl>	logFC <dbl>	avgExpr <dbl>
DDR1	DDR1	2.726390e-13	3.688108e-11	-8.2065100	0.15338875	9.738859
RFC2	RFC2	7.639613e-03	2.832300e-02	2.7045519	-0.16549110	4.826661
HSPA6	HSPA6	5.053565e-01	6.822451e-01	0.6676391	-0.03513683	5.174343
PAX8	PAX8	1.032228e-01	2.279058e-01	-1.6399110	0.04013541	7.693367
GUCA1A	GUCA1A	4.049680e-02	1.101945e-01	2.0672140	-0.11901233	5.350550
UBA7	UBA7	5.969303e-04	3.271467e-03	-3.5148444	0.11727136	6.392134

2.2.2 RNA-Seq Dataset: GSE153873

```
# Run t-test analysis using runTtest function with GSE153873 dataset
gse153873TtestRes <- runTtest(countMatrix = gse153873_count_normalized, groups =␣
↪as.factor(gse153873_anno$group))
# Show some first rows of the result tables
head(gse153873TtestRes)
```

A data.frame: 6 × 6

	SYMBOL <chr>	p.value <dbl>	pFDR <dbl>	statistic <dbl>	logFC <dbl>	avgExpr <dbl>
SGIP1	SGIP1	0.0012270882	0.02371747	-3.6010219	0.08215025	10.496064
NECAP2	NECAP2	0.6234944628	0.79397072	0.4963944	-0.01105260	8.536523
AZIN2	AZIN2	0.0034020925	0.03893733	-3.2730126	0.09684578	8.539012
AGBL4	AGBL4	0.0660431729	0.22281177	-1.9150867	0.12275431	7.322219
CLIC4	CLIC4	0.3607282878	0.58679331	-0.9294218	0.04476076	10.450300
SLC45A1	SLC45A1	0.0003813355	0.01386466	-4.0371559	0.12722385	8.026798

2.3 Differential Analysis using edgeR

```r
# Install edgeR package from Bioconductor
suppressMessages({
    if (!require("BiocManager", quietly = TRUE)) {
        install.packages("BiocManager")
    }
    suppressWarnings(BiocManager::install("edgeR"))
})
# Import edgeR package
suppressPackageStartupMessages({library("edgeR")})
```

```r
#' @description This function performs differential expression analysis using␣
↪edgeR
#'
#' @param countMatrix A matrix of gene expression data where rows represent␣
↪genes and columns represent samples.
#' @param groups A vector specifying the grouping of samples. Should have the␣
↪same length as the number of columns in countMatrix.
#' @return A dataframe containing the result of the analysis.

runEdgeR <- function(countMatrix, groups) {
    # Creates a DGEList object from a table of counts (rows=features,␣
↪columns=samples),
    # group indicator for each column, library size (optional) and a table of␣
↪feature annotation (optional).
    dge <- DGEList(counts = countMatrix, group = factor(groups))

    # Calculating the normalization factor
    dge <- calcNormFactors(object = dge)

    # Estimate dispersion
    dge <- suppressMessages(estimateDisp(y = dge))

    # Perform exact test
    et <- exactTest(object = dge)

    # Getting top DE genes
    top_degs <- topTags(object = et, n = "Inf")

    # Extract results and additional information
    top_degs$table$SYMBOL <- rownames(top_degs$table)
    top_degs$table$p.value <- top_degs$table$PValue
    top_degs$table$pFDR <- top_degs$table$FDR
    top_degs$table$statistic <- top_degs$table$logFC
    top_degs$table$avgExpr <- top_degs$table$logCPM
```

```r
    # Return the results with only important columns
    top_degs$table[, c("SYMBOL", "p.value", "pFDR", "statistic", "logFC",␣
↪"avgExpr")]
}
```

2.3.1 Microarray Dataset: GSE5281

```r
# Run differential analysis using runEdgeR function with GSE5281 dataset
gse5281EdgeRRes <- runEdgeR(countMatrix = gse5281_expr, groups = as.
↪factor(gse5281_anno$group))
head(gse5281EdgeRRes)
```

A data.frame: 6 × 6

	SYMBOL <chr>	p.value <dbl>	pFDR <dbl>	statistic <dbl>	logFC <dbl>	avgExpr <dbl>
SST	SST	7.080598e-10	1.570831e-05	-0.5171403	-0.5171403	6.147887
TMEM106A	TMEM106A	1.954961e-08	1.532170e-04	0.5808589	0.5808589	5.747591
MIR9-1HG	MIR9-1HG	3.084974e-08	1.532170e-04	0.4688537	0.4688537	6.185845
KCNE4	KCNE4	3.312364e-08	1.532170e-04	0.5520398	0.5520398	5.807757
HIF3A	HIF3A	3.854443e-08	1.532170e-04	0.5054603	0.5054603	5.976659
COL1A1	COL1A1	4.143799e-08	1.532170e-04	0.5985071	0.5985071	5.635229

2.3.2 RNA-Seq Dataset: GSE153873

```r
# Run differential analysis using runEdgeR function with GSE153873 dataset
gse153873EdgeRRes <- runEdgeR(countMatrix = gse153873_count, groups = as.
↪factor(gse153873_anno$group))
head(gse153873EdgeRRes)
```

A data.frame: 6 × 6

	SYMBOL <chr>	p.value <dbl>	pFDR <dbl>	statistic <dbl>	logFC <dbl>	avgExpr <dbl>
LOC101926975	LOC101926975	1.020689e-10	1.581608e-06	-1.992590	-1.992590	-0.3342804
GMNC	GMNC	1.534999e-10	1.581608e-06	2.284438	2.284438	-1.1354461
ADAMTS2	ADAMTS2	1.757713e-10	1.581608e-06	1.560794	1.560794	3.7462452
LOC100506136	LOC100506136	2.331465e-10	1.581608e-06	1.556556	1.556556	1.2503550
DNAH11	DNAH11	8.022113e-10	4.353601e-06	1.247874	1.247874	2.5744443
LOC100129316	LOC100129316	1.117520e-09	5.053985e-06	1.799734	1.799734	1.1501805

2.4 Differential Analysis using DESeq2

```r
# Installing and loading the library
suppressMessages({
    if (!require("BiocManager", quietly = TRUE)) {
        install.packages("BiocManager")
    }
    suppressWarnings(BiocManager::install("DESeq2", update = F))
```

```r
})

# Import DESeq2 package
suppressPackageStartupMessages({library("DESeq2")})
```

```r
#' @description This function performs differential expression analysis using␣
↪DESeq2
#'
#' @param countMatrix A matrix of gene expression data where rows represent␣
↪genes and columns represent samples.
#' @param groups A vector specifying the grouping of samples. It should have the␣
↪same length as the number of columns in countMatrix.
#' @return A dataframe containing the result of the analysis.

runDESeq2 <- function(countMatrix, groups) {
    # Constructing a group table that contains information about group members␣
↪for all patients.
    coldata <- data.frame(
        sample = colnames(countMatrix),
        condition = as.factor(groups),
        row.names = "sample"
    )
    # Suppressing messages to avoid printing DESeq2 messages
    suppressMessages({
        # Create a DESeqDataSet object
        dds <- DESeqDataSetFromMatrix(countData = round(countMatrix), colData =␣
↪coldata,
                                      design = ~condition)
        # Relevel the 'condition' factor
        dds$condition <- relevel(x = dds$condition, ref = "c")
        #  # Filter out low-count rows
        # dds <- dds[rowSums(counts(dds)) >= 10, ]
        # Run DESeq analysis
        dds <- suppressWarnings(DESeq(object = dds))
    })

    # Get names of results
    resultsNames(object = dds)

    # Get differential expression results
    res <- results(object = dds)

    # Drop na rows
    res <- res[complete.cases(res), ]
```

```
    # Order results by adjusted p-value
    res <- as.data.frame(res[order(res$padj), ])

    # Extract results and additional information
    res$SYMBOL <- rownames(res)
    res$p.value <- res$pvalue
    res$statistic <- res$stat
    res$logFC <- res$log2FoldChange
    res$avgExpr <- log2(res$baseMean + 1)
    res$pFDR <- p.adjust(res$p.value, method = "fdr")

    # Return the results with only important columns
    res[, c("SYMBOL", "p.value", "pFDR", "statistic", "logFC", "avgExpr")]
}
```

2.4.1 Microarray Dataset: GSE528

```
# Run DESeq2 analysis using runDESeq2 function with GSE5281 dataset
gse5281DESeq2Res <- runDESeq2(countMatrix = gse5281_expr, groups = as.
↪factor(gse5281_anno$group))
# Show some first rows of the result
head(gse5281DESeq2Res)
```

A data.frame: 6 × 6

	SYMBOL <chr>	p.value <dbl>	pFDR <dbl>	statistic <dbl>	logFC <dbl>	avgExpr <dbl>
SST	SST	1.129155e-08	0.0001970714	-5.710091	-0.5242242	2.971887
MIR9-1HG	MIR9-1HG	7.674145e-08	0.0006696842	5.374627	0.4835129	3.021356
SIMC1P1	SIMC1P1	3.085662e-07	0.0017951350	5.118138	0.4015684	3.259756
TMEM106A	TMEM106A	4.542612e-07	0.0019820551	5.044688	0.5967721	2.513752
HIF3A	HIF3A	7.322126e-07	0.0025558614	4.952619	0.5130156	2.786496
KCNE4	KCNE4	1.657230e-06	0.0048206066	4.791277	0.5523038	2.593759

2.4.2 RNA-Seq Dataset: GSE153873

```
# Run DESeq2 analysis using runDESeq2 function with GSE153873 dataset
gse153873DESeq2Res <- runDESeq2(countMatrix = gse153873_count, groups = as.
↪factor(gse153873_anno$group))
# Show some first rows of the result
head(gse153873DESeq2Res)
```

A data.frame: 6 × 6

	SYMBOL <chr>	p.value <dbl>	pFDR <dbl>	statistic <dbl>	logFC <dbl>	avgExpr <dbl>
NPC1L1	NPC1L1	5.347564e-12	1.147213e-07	6.896031	3.6836254	4.766138
TRAF3IP2-AS1	TRAF3IP2-AS1	4.105603e-11	2.935917e-07	6.600216	0.6036339	8.443113
TRAPPC1	TRAPPC1	3.229438e-11	2.935917e-07	-6.635710	-0.6230562	8.629783
LOC100506136	LOC100506136	7.284017e-11	3.906600e-07	6.514690	1.5676504	5.027996
MED29	MED29	2.622441e-10	1.125185e-06	-6.319598	-0.6422595	10.243159
LOC101926975	LOC101926975	3.774822e-10	1.349688e-06	-6.263065	-2.0326924	3.333696

2.5 Differential Analysis Result Visualization

```r
# Install and import the ggplot2 package
suppressWarnings({
    suppressMessages({
        install.packages("ggplot2")
        library(ggplot2)
    })
})
```

```
The downloaded binary packages are in
/var/folders/lz/5chptxdx2yg5vvszdt29pt400000gn/T//RtmpmTTyNe/downloaded_packages
```

2.5.1 MA Plot

```r
#' @description This function visualizes differential expression results with an␣
↪MA plot
#'
#' @param DEResult A dataframe containing differential expression results,␣
↪typically generated by a DE analysis function.
#' @param pThreshold A numerical value specifying the threshold of p-values to␣
↪filter DE genes (default: 0.05).
#' @param useFDR A boolean indicating whether to use adjusted p-values (False␣
↪Discovery Rate - FDR) for significance (default: TRUE).
#' @param logFCThreshold A numerical value specifying the threshold for absolute␣
↪log-fold change to consider genes as differentially expressed (default: 1).
#' @param labels A vector of gene labels for highlighting specific genes␣
↪(default: NULL).
#' @param fitMethod A character string specifying the method for fitting the␣
↪smooth curve in the MA plot ("loess" or "lowess", default: "loess").
#' @return A plot visualizing the differential expression results with an MA␣
↪plot.

plotMA <- function(DEResult, pThreshold = 0.05, useFDR = TRUE, logFCThreshold =␣
↪0.5, labels = NULL, fitMethod = "loess") {
    # Filter the DE genes for the plot
    plotDat <- data.frame(
```

```
        x = DEResult$avgExpr,
        y = DEResult$logFC,
        isSig = (
            if (useFDR) {
                DEResult$pFDR < pThreshold
            } else {
                DEResult$p.value < pThreshold
            }
        )
    )
    # assign color for each gene
    plotDat$color <- factor(plotDat$isSig * (abs(plotDat$y) > logFCThreshold) *␣
↪sign(plotDat$y), levels = c(1, -1, 0))

    if (!is.null(labels)) {
        plotDat$label <- labels[DEResult$PROBEID]
    }
    # Create the scatter plot using ggplot
    pl <- ggplot(plotDat, aes(x = .data$x, y = .data$y, color = .data$color)) +
        geom_point() +
        theme_bw() +
        theme_minimal() +
        theme(
            axis.line.x = element_blank(),
            axis.line.y = element_blank(),
        ) +
        scale_color_manual(
            values = c(
                "1" = "#B80F0A",
                "-1" = "#004F98",
                "0" = "darkgray"
            ),
            labels = c(
                "1" = paste0("Upregulated (", sum(plotDat$color == 1, na.rm =␣
↪TRUE), ")"),
                "-1" = paste0("Downregulated (", sum(plotDat$color == -1, na.rm␣
↪= TRUE), ")"),
                "0" = paste0("Not significant (", sum(plotDat$color == 0, na.rm␣
↪= TRUE), ")")
            ),
            guide = guide_legend(override.aes = list(size = 3), title =␣
↪"Significance")
        ) +
        theme(
            legend.position = "bottom"
        ) +
        labs(
```

```
            x = "Average expression",
            y = "Log2 fold change"
        ) +
        geom_hline(yintercept = -logFCThreshold, linetype = "dashed") +
        geom_hline(yintercept = logFCThreshold, linetype = "dashed")

    if (!is.null(labels)) {
        labelDat <- filter(plotDat, !is.na(.data$label))

        pl <- pl + geom_label_repel(
            labelDat,
            mapping = aes(x = .data$x, y = .data$y, label = .data$label),
            size = 3,
            segment.size = 0.75,
            segment.color = "#888888",
            color = "black",
            box.padding = 1,
            force = 3,
            point.size = NA
        )
    }
    # Create the fit curve for the data
    if (!is.null(fitMethod)) {
        pl <- pl + geom_smooth(
            formula = y ~ x,
            method = fitMethod,
            se = FALSE,
            color = "#228b22"
        ) +
            geom_hline(yintercept = 0, color = "#888888")
    }
    # return the plot
    pl

}
```

```
options(repr.plot.width = 6, repr.plot.height = 6)
# Create a MA plot for the limma result of the GSE5281 dataset
MA1 <- plotMA(DEResult = gse5281LimmaRes, pThreshold = 0.05, useFDR = TRUE,␣
↪logFCThreshold = 0.5) + ggtitle("GSE5281")
# Create a MA plot for the limma result of the GSE153873 dataset
MA2 <- plotMA(DEResult = gse153873DESeq2Res, pThreshold = 0.05, useFDR = TRUE,␣
↪logFCThreshold = 0.5) + ggtitle("GSE153873")

options(repr.plot.width = 12, repr.plot.height = 6)
# Combine the two plots using grid.arrange function
combinedMA <- gridExtra::grid.arrange(
```

```
    MA1 + scale_x_continuous(expand = c(0.01, 0.01)),
    MA2 + scale_x_continuous(expand = c(0.01, 0.01)),
    ncol = 2
)
combinedMA
```

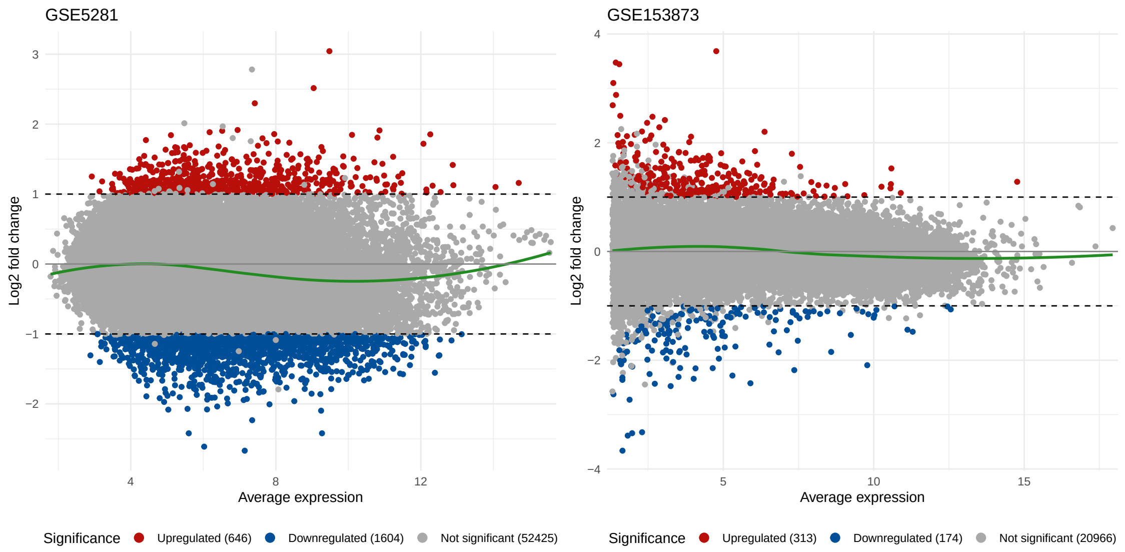

2.5.2 Volcano Plot

```
#' @description This function generates a volcano plot based on DE analysis␣
↪results
#'
#' @param DEResult A dataframe containing differential expression results,␣
↪typically generated by a DE analysis function.
#' @param pThreshold A numerical value specifying the threshold for p-values to␣
↪filter DE genes (default: 0.05).
#' @param useFDR A boolean indicating whether to use adjusted p-values (False␣
↪Discovery Rate - FDR) for significance (default: TRUE).
#' @param logFCThreshold A numerical value specifying the threshold for absolute␣
↪log-fold change to consider genes as differentially expressed (default: 1).
#' @return A filtered dataframe containing significant differential expression␣
↪results.
#' @examples
#' filtered_result <- filter_differential_expression(de_result, pThreshold = 0.
↪05, useFDR = TRUE, logFCThreshold = 0.5)

plotVolcanoDE <- function(DEResult, pThreshold = 0.05, useFDR = TRUE,␣
↪logFCThreshold = 0.5) {
```

```r
    # Check if the required columns exist in the DEResult
    if (!"logFC" %in% colnames(DEResult)) {
        stop("The logFC column is not in the results data frame.")
    }

    if (useFDR && !("pFDR" %in% colnames(DEResult))) {
        stop("The pFDR column is not in the results data frame.")
    } else if (!("p.value" %in% colnames(DEResult))) {
        stop("The p.value column is not in the results data frame.")
    }

    pvalues <- if (useFDR) {
        DEResult$pFDR
    } else {
        DEResult$p.value
    }
    # Filter out the DE Genes for plotting
    plotDat <- data.frame(
        x = DEResult$logFC,
        y = -log10(pvalues),
        color = ifelse(abs(DEResult$logFC) > logFCThreshold & pvalues <␣
↪pThreshold,  DEResult$logFC, NA)
    )

    isNoSig <- FALSE
    if (sum(is.na(plotDat$color)) == nrow(plotDat)) {
        isNoSig <- TRUE
        plotDat$color <- "gray"
    }
    # Create the plot
    pl <- ggplot(plotDat, aes(x = .data$x, y = .data$y, color = .data$color)) +
        geom_point() +
        geom_hline(yintercept = -log10(pThreshold), linetype = "dashed", color =␣
↪"black") +
        geom_vline(xintercept = -logFCThreshold, linetype = "dashed") +
        geom_vline(xintercept = logFCThreshold, linetype = "dashed") +
        labs(
            x = "log2 fold change",
            y = if (useFDR) {
                "-log10 pFDR"
            } else {
                "-log10 p-value"
            }
        ) +
        theme_bw() +
        theme(
```

```r
            panel.grid.minor = element_blank(),
            panel.background = element_blank(),
            axis.line.x = element_line(color = "darkgray"),
            axis.line.y = element_line(color = "darkgray"),
            plot.title = element_text(hjust = 0.5),
            legend.position = "none"
        )


    if (!isNoSig) {
        pl <- pl + scale_color_gradient(low = "blue", high = "red", na.value = 
 ↪"gray")
    } else {
        pl <- pl + scale_color_manual(values = "gray")
    }
    # Return the plot
    pl

}
```

```r
# Create a MA plot for the limma result of the GSE5281 dataset
Volplt1 <- plotVolcanoDE(DEResult = gse5281LimmaRes, pThreshold = 0.05, useFDR = 
 ↪TRUE, logFCThreshold = 0.5) + ggtitle("GSE5281")

# Create a MA plot for the limma result of the GSE153873 dataset
Volplt2 <- plotVolcanoDE(DEResult = gse153873DESeq2Res, pThreshold = 0.05, 
 ↪useFDR = TRUE, logFCThreshold = 0.5) + ggtitle("GSE153873")

# Adjust size of the plot
options(repr.plot.width = 12, repr.plot.height = 6)
# Combine the two plots using grid.arrange function
combinedVol <- gridExtra::grid.arrange(
    Volplt1 + scale_x_continuous(expand = c(0.01, 0.01)),
    Volplt2 + scale_x_continuous(expand = c(0.01, 0.01)),
    ncol = 2
)
combinedVol
```

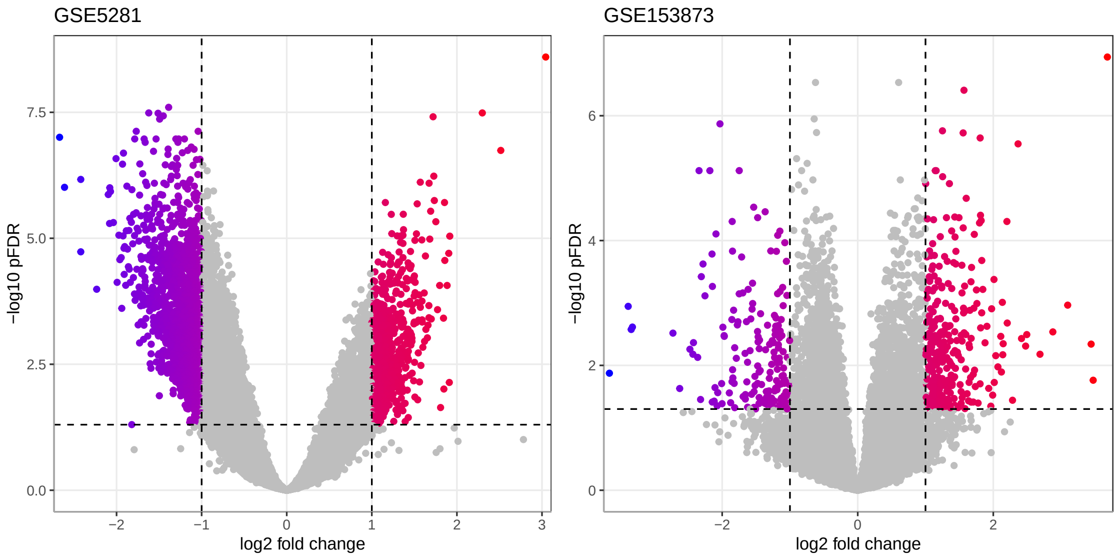

2.6 Gene-level Consensus Analysis

```
# Install and import packages
suppressMessages({
    suppressWarnings(if (!require("ggvenn")) install.packages("ggvenn"))
    suppressWarnings(if (!require("ggnewscale")) install.packages("ggnewscale"))
    suppressWarnings(if (!require("tidyr")) install.packages("tidyr"))

    suppressPackageStartupMessages({library(ggvenn)})
    suppressPackageStartupMessages({library(tidyr)})
    suppressPackageStartupMessages({library(ggnewscale)})
})
```

```
# Put all the result dataframes to a list for the plotVennDE function
gse5281DEResults <- list(gse5281LimmaRes, gse5281TtestRes, gse5281EdgeRRes,␣
 ↪gse5281DESeq2Res)
names(gse5281DEResults) <- c("limma", "t-test", "edgeR", "DESeq2")

# Put all the result dataframes to a list for the plotVennDE function
gse153873DEResults <- list(gse153873LimmaRes, gse153873TtestRes,␣
 ↪gse153873EdgeRRes, gse153873DESeq2Res)
names(gse153873DEResults) <- c("limma", "t-test", "edgeR", "DESeq2")
```

2.6.1 Gene Venn Diagram

```r
#' @ description This function generates a Venn diagram to visualize the overlap␣
↪of differentially expressed genes or features
#'                between multiple sets of results.
#'
#' @param aResults A list containing multiple sets of differential expression␣
↪analysis results.
#'                Each element in the list should be a data frame or a tibble␣
↪with columns representing
#'                differentially expressed genes or features and relevant␣
↪statistics.
#' @param pThreshold A numerical value specifying the threshold for filtering␣
↪differentially expressed genes (default: 0.05).
#' @param useFDR A logical value indicating whether to use normal p-value or FDR␣
↪p-value to filter out the DE genes (default: TRUE).
#' @param stat A character string specifying the statistical metric to use for␣
↪filtering genes (default: "logFC" for log-fold change).
#' @param statThreshold A numerical value specifying the threshold value for the␣
↪chosen statistical metric to filter differentially expressed genes (default:␣
↪0).
#'
#' @return A Venn diagram displaying the overlap of differentially expressed␣
↪genes or features among the specified result sets.

plotVenn <- function(aResults, pThreshold = 0.05, useFDR = TRUE, stat = "logFC",␣
↪statThreshold = 0) {
    # Check the input data requirements
    if (length(aResults) < 2) {
        stop("The number of DE results must be at least 2.")
    }

    for (Res in aResults) {
        if (useFDR && !("pFDR" %in% colnames(Res))) {
            stop("The F DR adjusted p-value column is not in the results data␣
↪frame.")
        } else {
            if (!("p.value" %in% colnames(Res))) {
                stop("The p.value column is not in the results data frame.")
            }
        }

        if (!stat %in% colnames(Res)) {
            stop("The statistic column is not in the results data frame.")
        }
    }
```

```
    # Filter out DE Genes for the plot
    plotDat <- lapply(aResults, function(Res) {
            filtered_df <- filter(data.frame(Res),
                abs(.data[[stat]]) > statThreshold & (
                    if (useFDR) {
                        .data$pFDR < pThreshold
                    } else {
                        .data$p.value < pThreshold
                    }
                )
            )
            filtered_df[["SYMBOL"]]
    })

    if (is.null(names(plotDat))) {
        names(plotDat) <- paste0("Dataset ", seq_along(plotDat))
    }
    pR <- ggvenn::ggvenn(plotDat,
           fill_color = c(
               "#316b9d", "#f77a65",
               "#a6a1d0", "#fea9c4",
               "#74e7bc", "#febb73",
               "#1db4db", "#ffc5a6",
               "#b6c9fa", "#ee5437"),
           stroke_size = 0.5,
           set_name_size = 4,
           fill_alpha = 0.75
    )
    # Return the plot
    return(pR)
}
```

```
options(repr.plot.width = 12, repr.plot.height = 6)

# Create a venn diagram for the microarray result
Vennplt1 <- plotVenn(aResults = gse5281DEResults, pThreshold = 0.05, useFDR =␣
↪TRUE, stat = "logFC", statThreshold = 0)
Vennplt1 <- Vennplt1 + ggtitle("GSE5281")
# Create a venn diagram for the RNA-seq result
Vennplt2 <- plotVenn(aResults = gse153873DEResults, pThreshold = 0.05, useFDR =␣
↪TRUE, stat = "logFC", statThreshold = 0)
Vennplt2 <- Vennplt2 + ggtitle("GSE153873")
# Adjust size of the plot
options(repr.plot.width = 14, repr.plot.height = 7)
# Combine the two plots using grid.arrange function
combinedVenn <- gridExtra::grid.arrange(
    Vennplt1,
```

```
    Vennplt2,
    ncol = 2
)
combinedVenn
```

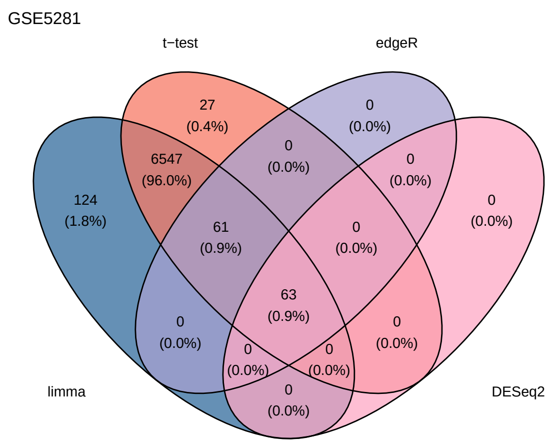

2.6.2 Gene Heatmap Plot

```
#' @description Generates a heatmap for differentially expressed genes (DEGs)␣
↪based on log-fold change and p-values.
#'
#' @param DEResults A list of at least length two containing DE analysis results␣
↪for multiple datasets.
#' @param numGenes An integer specifying the number of common genes to plot
#' @param useFDR A logical value indicating whether to use normal p-value or FDR␣
↪p-value to filter out the DE genes (default: TRUE).#' @param labels A named␣
↪vector of labels for genes. Default is `NULL`.
#' @param logFCLims A numeric vector of length 2 specifying the limits for␣
↪log-fold change. Default is c(-5, 5).
#' @param negLog10pValueLims A numeric vector of length 2 specifying the limits␣
↪for negative log10 p-value. Default is c(0, 5).
#' @return A ggplot object representing the heatmap of DEGs.

plotDEGeneHeatmap <- function(DEResults, numGenes = 30, useFDR = TRUE, logFCLims␣
↪= c(-5, 5), negLog10pValueLims = c(0, 5)) {
    commonGenes <- lapply(DEResults, function(x) x$SYMBOL)
    commonGenes <- Reduce(intersect, commonGenes)[1:numGenes]
    if (any(commonGenes == 0)) {
```

```
        stop("No common genes found between the input genes and the genes in the␣
↪DE results")
    }

    DEdfs <- lapply(DEResults, function(x) x[match(commonGenes, x$SYMBOL),])

    labels <- commonGenes

    scaleMinMax <- function(x, minx, maxx) {
        x[x < minx] <- minx
        x[x > maxx] <- maxx
        x
    }

    if (is.null(names(DEdfs))) {
        names(DEdfs) <- paste0("Dataset ", seq_along(DEdfs))
    }

    plotData <- lapply(names(DEdfs), function(n) {
            DEdf <- as.data.frame(DEdfs[[n]])
            DEdf$p.value <- scaleMinMax(abs(log10(ifelse(rep(useFDR,␣
↪nrow(DEdf)), DEdf$pFDR, DEdf$p.value))), negLog10pValueLims[1],␣
↪negLog10pValueLims[2])
            DEdf$logFC  <-scaleMinMax(DEdf$logFC, logFCLims[1], logFCLims[2])
            DEdf$label <- factor(labels, levels = labels)
            DEdf$dataset <- n
            DEdf <- DEdf[, c("label", "logFC", "p.value", "dataset")]
            DEdf

        })
    plotData <- do.call(plotData, what = rbind)
    plotData <- gather(plotData, "type", "value", -"label", -"dataset")
    plotData$label <- factor(plotData$label, levels = labels)
    plotData$dataset <- factor(plotData$dataset, levels = names(DEdfs))
    plotData$type <- factor(plotData$type, levels = c("p.value", "logFC"))
    plotData$colOrder <- as.numeric(plotData$dataset) + as.
↪numeric(plotData$type)*length(DEdfs) + as.numeric(plotData$dataset)*0.01 + as.
↪numeric(plotData$type)*0.1

    uniqueY <- sort(unique(plotData$colOrder))

    x <- ggplot() +
        geom_tile(data = plotData[plotData$type == "logFC",], aes(x = .
↪data$label, y = .data$colOrder, fill = .data$value, width = 1, height = 1)) +
        scale_fill_gradient2(
            high = "#B80F0A",
            low = "#004F98",
```

```
            mid = "white",
            na.value = "white",
            limits = logFCLims,
        ) +
        labs(fill = "log2 FC") +

        new_scale_fill() +
        geom_tile(data = plotData[plotData$type == "p.value",], aes(x = .
↪data$label, y = .data$colOrder, fill = .data$value, width = 1, height = 1)) +
        scale_fill_gradient(
            low = "white",
            high = "#B80F0A",
            na.value = "white",
            limits = negLog10pValueLims,
            guide = guide_colorbar(title = paste0("-log10", ifelse(useFDR, "␣
↪pFDR", " p-value")))
        ) +
        theme_minimal() +
        coord_flip() +
        theme(
            axis.title.y = element_blank(),
            axis.title.x = element_blank(),
            axis.text.x.bottom = element_text(angle = 45, vjust = 1, hjust = 1),
            panel.grid.major = element_blank(),
            panel.grid.minor = element_blank()
        ) +
        scale_x_discrete(
            labels = labels
        ) +
        scale_y_continuous(
            breaks = uniqueY,
            labels = rep(names(DEdfs), 2),
            expand = c(0, 0),
            sec.axis = sec_axis(~., breaks = sapply(seq_along(c(
              paste0("-log10", ifelse(useFDR, " pFDR", " p-value")),
              "log2 FC"
            )), function(i) mean(uniqueY[(i-1)*length(DEdfs) + 1:
↪length(DEdfs)])), labels = c(
              paste0("-log10", ifelse(useFDR, " pFDR", " p-value")),
              "log2 FC"
            ))
        )

    return(x)
}
```

```r
options(repr.plot.width = 12, repr.plot.height = 6)

# Create a heat map for DE analysis results
heatmap1 <- plotDEGeneHeatmap(gse5281DEResults, numGenes = 30, useFDR = TRUE, logFCLims = c(-5, 5), negLog10pValueLims = c(0, 5))
heatmap1 <- heatmap1 + ggtitle("GSE5281")
# Create a heat map for DE analysis results
heatmap2 <- plotDEGeneHeatmap(gse153873DEResults, numGenes = 30, useFDR = TRUE, logFCLims = c(-5, 5), negLog10pValueLims = c(0, 5))
heatmap2 <- heatmap2 + ggtitle("GSE153873")
# Adjust size of the plot
options(repr.plot.width = 14, repr.plot.height = 7)
# Combine the two plots using grid.arrange function
combinedPlt1 <- gridExtra::grid.arrange(
    heatmap1,
    heatmap2,
    ncol = 2
)
combinedPlt1
```

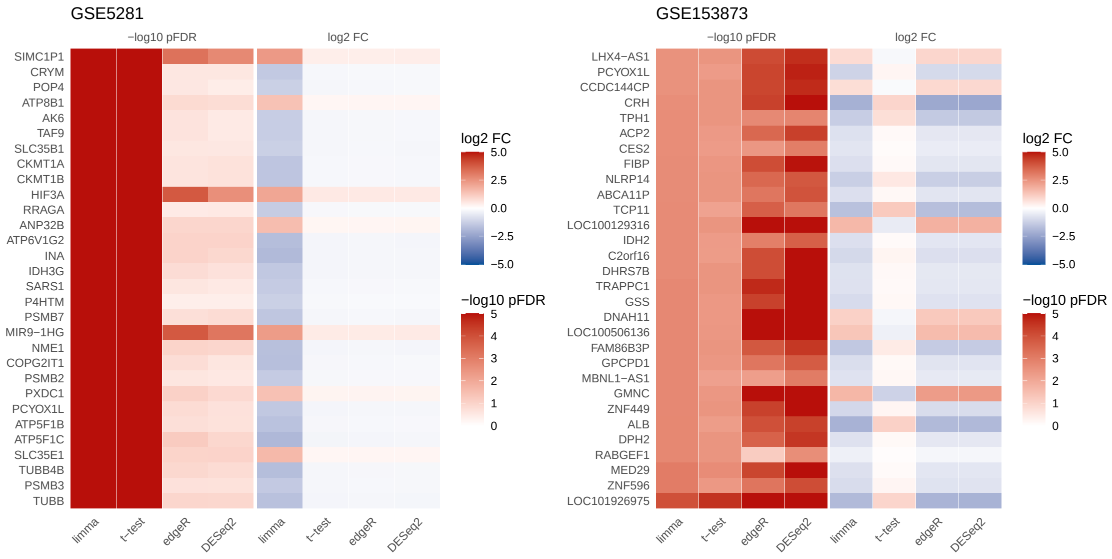

3 Submodule 03: Processing Pathway Information

```r
# Install the magick package for the ReactomeContentService4R
system("sudo apt-get install -y libmagick++-dev", intern = TRUE, ignore.stdout = TRUE)
# Install and import packages
suppressWarnings({
```

```
    suppressMessages({
        BiocManager::install(c("topGO", "org.Hs.eg.db", "KEGGREST",␣
↪"ReactomeContentService4R"), update = F)
        library(topGO)
        library(org.Hs.eg.db)
        library(ReactomeContentService4R)
    })
})
```

3.1 Retrieving GO Terms

```
# Get all gene SYMBOLS in the org.Hs.eg.db database
allGeneSymbols <- keys(org.Hs.eg.db, keytype = "SYMBOL")
# Create a numeric vector for the `allGenes` argument for the new function
geneList <- 1:length(allGeneSymbols)
# Assign all gene SYMBOLS as names of the numeric vector
names(geneList) <- allGeneSymbols

# Retrieve all the GO terms related to the gene list obtained from the␣
↪expression matrix
GOdata <- new("topGOdata", description = "", ontology = "BP",
              allGenes = geneList, geneSel = function(x) x, nodeSize = 10,
              annot = annFUN.org, ID = "alias", mapping = "org.Hs.eg")
# Obtain a list of genes for each GO term
allGO = genesInTerm(GOdata)
# Show the first 5 GO terms
allGO[1:5]
```

```
Building most specific GOs .....

        ( 12399 GO terms found. )


Build GO DAG topology ..........

        ( 15709 GO terms and 35427 relations. )


Annotating nodes ...............

        ( 19099 genes annotated to the GO terms. )
```

$‘GO:0000002‘ 1. ’AKT3’ 2. ’ATXN8’ 3. ’BHD’ 4. ’DNA2’ 5. ’DNAJA3’ 6. ’ENDOG’ 7. ’FLCN’ 8. ’LIG3’ 9. ’LONP1’ 10. ’MDP1’ 11. ’MEF2A’ 12. ’METTL4’ 13. ’MGME1’ 14. ’MPV17’ 15. ’OPA1’ 16. ’PARP1’ 17. ’PIF1’ 18. ’PIM1’ 19. ’POLB’ 20. ’POLG’ 21. ’POLG2’ 22. ’PPARGC1A’ 23. ’PRIMPOL’ 24. ’RRM1’ 25. ’RRM2B’ 26. ’SESN2’ 27. ’SLC25A33’

28. 'SLC25A36' 29. 'SLC25A4' 30. 'SSBP1' 31. 'STOX1' 32. 'TOP3A' 33. 'TP53' 34. 'TWNK' 35. 'TYMP'

$'GO:0000003' 1. 'A1CF' 2. 'A2M' 3. 'AAAS' 4. 'AAT1' 5. 'ABAT' 6. 'ABCC8' 7. 'ABHD2' 8. 'ACE' 9. 'ACE2' 10. 'ACF' 11. 'ACOD1' 12. 'ACOX1' 13. 'ACR' 14. 'ACRBP' 15. 'ACRV1' 16. 'ACSBG2' 17. 'ACSL4' 18. 'ACTL7A' 19. 'ACTL9' 20. 'ACTR2' 21. 'ACTR3' 22. 'ACVR1' 23. 'ACVR1B' 24. 'ACVR1C' 25. 'ACVR2A' 26. 'ADA' 27. 'ADAD1' 28. 'ADAD2' 29. 'ADAM15' 30. 'ADAM18' 31. 'ADAM2' 32. 'ADAM20' 33. 'ADAM21' 34. 'ADAM28' 35. 'ADAM29' 36. 'ADAM30' 37. 'ADAM32' 38. 'ADAMTS1' 39. 'ADAMTS16' 40. 'ADAMTS2' 41. 'ADCY10' 42. 'ADCY3' 43. 'ADCY7' 44. 'AD-CYAP1' 45. 'ADCYAP1R1' 46. 'ADGB' 47. 'ADGRG1' 48. 'ADGRG2' 49. 'ADIG' 50. 'ADM' 51. 'ADNP' 52. 'ADRA2A' 53. 'ADRA2B' 54. 'AFF4' 55. 'AFG2A' 56. 'AFP' 57. 'AGFG1' 58. 'AGO2' 59. 'AGO4' 60. 'AGRP' 61. 'AHRR' 62. 'AKAP3' 63. 'AKAP4' 64. 'AKR1C3' 65. 'AKT1' 66. 'ALDOA' 67. 'ALKBH5' 68. 'ALOX15B' 69. 'ALPL' 70. 'AMBP' 71. 'AMD1' 72. 'AMH' 73. 'AMHR2' 74. 'ANAPC1' 75. 'ANAPC10' 76. 'ANAPC11' 77. 'ANAPC13' 78. 'ANAPC15' 79. 'ANAPC16' 80. 'ANAPC2' 81. 'ANAPC4' 82. 'ANAPC5' 83. 'ANAPC7' 84. 'ANCR' 85. 'ANG' 86. 'ANGPT2' 87. 'ANKLE1' 88. 'ANKRD31' 89. 'ANKRD49' 90. 'ANTXR1' 91. 'ANXA1' 92. 'AP3B1' 93. 'APC2' 94. 'APELA' 95. 'APLF' 96. 'APOB' 97. 'APOL2' 98. 'APOL3' 99. 'APP' 100. 'AR' 101. 'AREG' 102. 'ARHGDIB' 103. 'ARID4A' 104. 'ARID4B' 105. 'ARID5B' 106. 'ARMC12' 107. 'ARMC2' 108. 'ASB1' 109. 'ASF1B' 110. 'ASH1L' 111. 'ASIP' 112. 'ASPM' 113. 'ASTL' 114. 'ASZ1' 115. 'ATAT1' 116. 'ATD' 117. 'ATM' 118. 'ATN1' 119. 'ATP1A1' 120. 'ATP1A4' 121. 'ATP2B2' 122. 'ATP2B4' 123. 'ATP7A' 124. 'ATP8B3' 125. 'ATR' 126. 'ATRX' 127. 'AURKA' 128. 'AURKC' 129. 'AVP' 130. 'AVPR1A' 131. 'AXDND1' 132. 'AXIN1' 133. 'AXL' 134. 'AZI2' 135. 'AZIN2' 136. 'B4GALNT1' 137. 'B4GALT1' 138. 'BACH1' 139. 'BAG6' 140. 'BAK1' 141. 'BAP1' 142. 'BASP1' 143. 'BAX' 144. 'BBS1' 145. 'BBS2' 146. 'BBS4' 147. 'BCAP31' 148. 'BCL2' 149. 'BCL2L1' 150. 'BCL2L10' 151. 'BCL2L11' 152. 'BCL2L2' 153. 'BCL5' 154. 'BCL6' 155. 'BIK' 156. 'BIRC3' 157. 'BMAL1' 158. 'BMP15' 159. 'BMP4' 160. 'BMP5' 161. 'BMP6' 162. 'BMP7' 163. 'BMPR1A' 164. 'BMPR1B' 165. 'BMPR2' 166. 'BNC1' 167. 'BOK' 168. 'BOLL' 169. 'BPY2' 170. 'BPY2B' 171. 'BPY2C' 172. 'BRCA2' 173. 'BRD2' 174. 'BRDT' 175. 'BRINP1' 176. 'BRIP1' 177. 'BRME1' 178. 'BSG' 179. 'BSPH1' 180. 'BTBD18' 181. 'BUB3' 182. 'C14orf39' 183. 'C16orf92' 184. 'C1orf146' 185. 'C1QBP' 186. 'C2CD6' 187. 'C3' 188. 'C3orf62' 189. 'C9orf78' 190. 'CA12' 191. 'CABS1' 192. 'CABYR' 193. 'CACNA1H' 194. 'CAD' 195. 'CADM1' 196. 'CALCA' 197. 'CALR' 198. 'CALR3' 199. 'CAPN2' 200. 'CASP2' 201. · 202. 'TEP1' 203. 'TERB1' 204. 'TERB2' 205. 'TERF1' 206. 'TESC' 207. 'TESK1' 208. 'TESK2' 209. 'TESMIN' 210. 'TEX101' 211. 'TEX11' 212. 'TEX12' 213. 'TEX14' 214. 'TEX15' 215. 'TEX19' 216. 'TFAP2C' 217. 'TFPT' 218. 'TFQTL2' 219. 'TGFB2' 220. 'TGFB3' 221. 'TGFBR1' 222. 'TGS1' 223. 'TH' 224. 'THBD' 225. 'THRA' 226. 'THRB' 227. 'TIAL1' 228. 'TIFAB' 229. 'TIMP1' 230. 'TIPARP' 231. 'TLE6' 232. 'TLR3' 233. 'TLR9' 234. 'TMED2' 235. 'TMEM119' 236. 'TMEM203' 237. 'TMEM95' 238. 'TMF1' 239. 'TMPRSS12' 240. 'TNC' 241. 'TNFAIP6' 242. 'TNFSF10' 243. 'TNP1' 244. 'TNP2' 245. 'TOB2' 246. 'TOP2A' 247. 'TOP2B' 248. 'TOP3A' 249. 'TOP6BL' 250. 'TOPAZ1' 251. 'TP63' 252. 'TPGS1' 253. 'TPPP2' 254. 'TPPP3' 255. 'TRAC' 256. 'TRAP' 257. 'TRIM27' 258. 'TRIM28' 259. 'TRIM36' 260. 'TRIM75' 261. 'TRIP13' 262. 'TRO' 263. 'TRPC3' 264. 'TRPC6' 265. 'TRPC7' 266. 'TSGA10' 267. 'TSNAX' 268. 'TSNAXIP1' 269. 'TSPAN8' 270. 'TSPY1' 271. 'TSPY10' 272. 'TSPY2' 273. 'TSPY3' 274. 'TSPY4' 275. 'TSPY8' 276. 'TSPY9' 277. 'TSSK1B' 278. 'TSSK2' 279. 'TSSK3' 280. 'TSSK4' 281. 'TSSK6' 282. 'TTC12'

283. 'TTC21A' 284. 'TTF1' 285. 'TTK' 286. 'TTLL1' 287. 'TTLL3' 288. 'TTLL8' 289. 'TTLL9' 290. 'TUBA8' 291. 'TUBB8' 292. 'TUBG1' 293. 'TUBG2' 294. 'TUBGCP2' 295. 'TUBGCP3' 296. 'TUBGCP4' 297. 'TUBGCP5' 298. 'TUBGCP6' 299. 'TUT4' 300. 'TUT7' 301. 'TXNDC2' 302. 'TXNDC8' 303. 'TXNRD3' 304. 'TYRO3' 305. 'UBAP2L' 306. 'UBB' 307. 'UBE2B' 308. 'UBE2J1' 309. 'UBE2Q1' 310. 'UBE3A' 311. 'UBR2' 312. 'UBTFL1' 313. 'UBXN8' 314. 'UCHL1' 315. 'UCN' 316. 'UCP2' 317. 'UMODL1' 318. 'UMPS' 319. 'UNC13B' 320. 'UNC5C' 321. 'UPF3A' 322. 'UPRT' 323. 'USP17L2' 324. 'USP26' 325. 'USP42' 326. 'USP9X' 327. 'USP9Y' 328. 'UTF1' 329. 'UTP14C' 330. 'VCX' 331. 'VDAC2' 332. 'VDAC3' 333. 'VDR' 334. 'VEGFA' 335. 'VGF' 336. 'VIP' 337. 'VIPAS39' 338. 'VMP1' 339. 'VPS13A' 340. 'VPS13B' 341. 'VPS54' 342. 'WASHC5' 343. 'WBP2NL' 344. 'WDR19' 345. 'WDR33' 346. 'WDR48' 347. 'WDR77' 348. 'WEE2' 349. 'WFDC2' 350. 'WIPF3' 351. 'WNT2B' 352. 'WNT3' 353. 'WNT4' 354. 'WNT5A' 355. 'WNT7A' 356. 'WNT9B' 357. 'WT1' 358. 'XDH' 359. 'XKRY' 360. 'XRCC2' 361. 'XRN2' 362. 'YBX2' 363. 'YBX3' 364. 'YTHDC1' 365. 'YTHDC2' 366. 'YTHDF2' 367. 'YTHDF3' 368. 'YY1' 369. 'ZAN' 370. 'ZAR1' 371. 'ZAR1L' 372. 'ZBTB16' 373. 'ZCWPW1' 374. 'ZDBF2' 375. 'ZFP41' 376. 'ZFP42' 377. 'ZFP57' 378. 'ZFPM2' 379. 'ZFX' 380. 'ZGLP1' 381. 'ZMIZ1' 382. 'ZMYND15' 383. 'ZNF148' 384. 'ZNF225' 385. 'ZNF296' 386. 'ZNF318' 387. 'ZNF32' 388. 'ZNF35' 389. 'ZNF449' 390. 'ZNF541' 391. 'ZNF628' 392. 'ZNF830' 393. 'ZP1' 394. 'ZP2' 395. 'ZP3' 396. 'ZP4' 397. 'ZPBP' 398. 'ZPBP2' 399. 'ZSCAN2' 400. 'ZSCAN21' 401. 'ZW10'

$‘GO:0000012‘ 1. 'APLF' 2. 'APTX' 3. 'ERCC6' 4. 'ERCC8' 5. 'LIG4' 6. 'PARP1' 7. 'SIRT1' 8. 'TDP1' 9. 'TERF2' 10. 'TNP1' 11. 'XNDC1N' 12. 'XRCC1'

$‘GO:0000018‘ 1. 'ABL1' 2. 'ACTB' 3. 'ACTL6A' 4. 'ACTR2' 5. 'ALYREF' 6. 'ANKLE1' 7. 'APLF' 8. 'ARID2' 9. 'ATAD5' 10. 'BCL5' 11. 'BCL6' 12. 'BLM' 13. 'BRD8' 14. 'CD28' 15. 'CD40' 16. 'CFL1' 17. 'CGAS' 18. 'CHEK1' 19. 'CLC' 20. 'CLCF1' 21. 'DMAP1' 22. 'EAF2' 23. 'EP400' 24. 'EPC1' 25. 'EPC2' 26. 'ERCC2' 27. 'ERCC6' 28. 'EXOSC3' 29. 'EXOSC6' 30. 'FANCB' 31. 'FBH1' 32. 'FIGNL1' 33. 'FOXP3' 34. 'FUS' 35. 'H1-0' 36. 'H1-1' 37. 'H1-10' 38. 'H1-2' 39. 'H1-3' 40. 'H1-4' 41. 'H1-5' 42. 'H1-6' 43. 'H1-7' 44. 'H1-8' 45. 'H1-9P' 46. 'HDGFL2' 47. 'HELB' 48. 'HELQ' 49. 'HMCES' 50. 'IL10' 51. 'IL2' 52. 'IL27RA' 53. 'IL4' 54. 'IL7R' 55. 'ING2' 56. 'ING3' 57. 'KAT5' 58. 'KDM1A' 59. 'KHDC3L' 60. 'KLHL15' 61. 'KMT5A' 62. 'KMT5B' 63. 'KMT5C' 64. 'KPNA1' 65. 'KPNA2' 66. 'LAP' 67. 'MAD2L2' 68. 'MAGEF1' 69. 'MBTD1' 70. 'MEAF6' 71. 'MLH1' 72. 'MMS19' 73. 'MORF4L1' 74. 'MORF4L2' 75. 'MRE11' 76. 'MRGBP' 77. 'MRNIP' 78. 'MSH2' 79. 'MSH3' 80. 'MSH6' 81. 'NDFIP1' 82. 'NSD2' 83. 'OOEP' 84. 'PARP1' 85. 'PARP3' 86. 'PARPBP' 87. 'PAXIP1' 88. 'PIAS4' 89. 'PMS2' 90. 'PMS2CL' 91. 'POLQ' 92. 'PPP4C' 93. 'PPP4R2' 94. 'PRDM7' 95. 'PRDM9' 96. 'PTPRC' 97. 'RAD50' 98. 'RAD51' 99. 'RAD51AP1' 100. 'RADX' 101. 'RECQL5' 102. 'RIF1' 103. 'RMI2' 104. 'RPA2' 105. 'RTEL1' 106. 'RUVBL1' 107. 'RUVBL2' 108. 'SENP3' 109. 'SETD2' 110. 'SHLD1' 111. 'SHLD2' 112. 'SHLD3' 113. 'SIRT6' 114. 'SLC15A4' 115. 'SMAP2' 116. 'SMARCAD1' 117. 'SMCHD1' 118. 'SPIDR' 119. 'STAT6' 120. 'SUPT6H' 121. 'TBX21' 122. 'TERF2' 123. 'TERF2IP' 124. 'TEX15' 125. 'TFRC' 126. 'TGFB1' 127. 'THOC1' 128. 'TIMELESS' 129. 'TNFSF13' 130. 'TNFSF4' 131. 'TP53BP1' 132. 'TRRAP' 133. 'UBE2B' 134. 'UBQLN4' 135. 'USP51' 136. 'VPS72' 137. 'WAS' 138. 'WDR48' 139. 'WRAP53' 140. 'YEATS4' 141. 'ZCWPW1' 142. 'ZNF365' 143. 'ZRANB3' 144. 'ZSCAN4'

$‘GO:0000022‘ 1. 'AURKB' 2. 'AURKC' 3. 'BIRC5' 4. 'CDCA8' 5. 'INCENP' 6. 'KIF23' 7. 'KIF4A' 8. 'KIF4B' 9. 'MAP10' 10. 'NUMA1' 11. 'PRC1' 12. 'RACGAP1'

3.2 Retrieving Pathways from KEGG Database

```
# Get all KEGG pathway IDs for human
pathwayList <- KEGGREST::keggList("pathway", "hsa")
# Query pathway data using pathway IDs
KEGGGeneSets <- lapply(names(pathwayList), function(pwId) {
  genes <- KEGGREST::keggGet(pwId)[[1]]$GENE
  if (is.null(genes)) return(NULL)
  gsub(";.*$", "", genes[seq_len(length(genes) / 2) * 2])})
names(KEGGGeneSets) <- names(pathwayList)

# Keep pathways that have more 10 genes
KEGGGeneSets <- KEGGGeneSets[sapply(KEGGGeneSets, function(x) length(x) >= 10)]
# Retrieve all the selected pathways
KEGGPathways <- pathwayList[names(KEGGGeneSets)]
# Show the first 5 KEGG pathways
KEGGGeneSets[1:5]
```

$hsa00010 1. 'HK3' 2. 'HK1' 3. 'HK2' 4. 'HKDC1' 5. 'GCK' 6. 'GPI' 7. 'PFKM' 8. 'PFKP' 9. 'PFKL' 10. 'FBP1' 11. 'FBP2' 12. 'ALDOC' 13. 'ALDOA' 14. 'ALDOB' 15. 'TPI1' 16. 'GAPDH' 17. 'GAPDHS' 18. 'PGK2' 19. 'PGK1' 20. 'PGAM1' 21. 'PGAM2' 22. 'PGAM4' 23. 'ENO3' 24. 'ENO2' 25. 'ENO1' 26. 'ENO4' 27. 'PKM' 28. 'PKLR' 29. 'PDHA2' 30. 'PDHA1' 31. 'PDHB' 32. 'DLAT' 33. 'DLD' 34. 'LDHAL6A' 35. 'LDHAL6B' 36. 'LDHA' 37. 'LDHB' 38. 'LDHC' 39. 'ADH1A' 40. 'ADH1B' 41. 'ADH1C' 42. 'ADH7' 43. 'ADH4' 44. 'ADH5' 45. 'ADH6' 46. 'AKR1A1' 47. 'ALDH2' 48. 'ALDH3A2' 49. 'ALDH1B1' 50. 'ALDH7A1' 51. 'ALDH9A1' 52. 'ALDH3B1' 53. 'ALDH3B2' 54. 'ALDH3A1' 55. 'ACSS1' 56. 'ACSS2' 57. 'GALM' 58. 'PGM1' 59. 'PGM2' 60. 'G6PC1' 61. 'G6PC2' 62. 'G6PC3' 63. 'ADPGK' 64. 'BPGM' 65. 'MINPP1' 66. 'PCK1' 67. 'PCK2'

$hsa00020 1. 'CS' 2. 'ACLY' 3. 'ACO2' 4. 'ACO1' 5. 'IDH1' 6. 'IDH2' 7. 'IDH3B' 8. 'IDH3G' 9. 'IDH3A' 10. 'OGDHL' 11. 'OGDH' 12. 'DLST' 13. 'DLD' 14. 'SUCLG1' 15. 'SUCLG2' 16. 'SUCLA2' 17. 'SDHA' 18. 'SDHB' 19. 'SDHC' 20. 'SDHD' 21. 'FH' 22. 'MDH1' 23. 'MDH2' 24. 'PC' 25. 'PCK1' 26. 'PCK2' 27. 'PDHA2' 28. 'PDHA1' 29. 'PDHB' 30. 'DLAT'

$hsa00030 1. 'GPI' 2. 'G6PD' 3. 'PGLS' 4. 'H6PD' 5. 'PGD' 6. 'RPE' 7. 'RPEL1' 8. 'TKT' 9. 'TKTL2' 10. 'TKTL1' 11. 'TALDO1' 12. 'RPIA' 13. 'SHPK' 14. 'DERA' 15. 'RBKS' 16. 'PGM1' 17. 'PGM2' 18. 'PRPS1L1' 19. 'PRPS2' 20. 'PRPS1' 21. 'RGN' 22. 'IDNK' 23. 'GLYCTK' 24. 'ALDOC' 25. 'ALDOA' 26. 'ALDOB' 27. 'FBP1' 28. 'FBP2' 29. 'PFKM' 30. 'PFKP' 31. 'PFKL'

$hsa00040 1. 'GUSB' 2. 'KL' 3. 'UGT2A1' 4. 'UGT2A3' 5. 'UGT2B17' 6. 'UGT2B11' 7. 'UGT2B28' 8. 'UGT1A6' 9. 'UGT1A4' 10. 'UGT1A1' 11. 'UGT1A3' 12. 'UGT2B10' 13. 'UGT1A9' 14. 'UGT2B7' 15. 'UGT1A10' 16. 'UGT1A8' 17. 'UGT1A5' 18. 'UGT2B15' 19. 'UGT1A7' 20. 'UGT2B4' 21. 'UGT2A2' 22. 'UGDH' 23. 'UGP2' 24. 'AKR1A1' 25. 'CRYL1' 26. 'RPE' 27. 'RPEL1' 28. 'XYLB' 29. 'AKR1B1' 30. 'AKR1B10' 31. 'AKR1B15' 32. 'DCXR' 33. 'SORD' 34. 'DHDH' 35. 'FGGY' 36. 'CRPPA'

$hsa00051 1. 'MPI' 2. 'PMM2' 3. 'PMM1' 4. 'GMPPB' 5. 'GMPPA' 6. 'GMDS' 7. 'GFUS' 8. 'FPGT' 9. 'FCSK' 10. 'ENOSF1' 11. 'HK3' 12. 'HK1' 13. 'HK2' 14. 'HKDC1' 15. 'PFKM' 16. 'PFKP' 17. 'PFKL' 18. 'FBP1' 19. 'FBP2' 20. 'PFKFB1' 21. 'PFKFB2'

22. 'PFKFB3' 23. 'PFKFB4' 24. 'TIGAR' 25. 'KHK' 26. 'SORD' 27. 'AKR1B1' 28. 'AKR1B10' 29. 'AKR1B15' 30. 'ALDOC' 31. 'ALDOA' 32. 'ALDOB' 33. 'TPI1' 34. 'TKFC'

3.3 Retrieving Pathways from REACTOME Database

```
# Retrieve the pathways information using the getSchemaClass function
pathways <- getSchemaClass(class = "Pathway", species = "human", all = TRUE)
# Get all pathway IDs to a vector
pathwayList <- as.character(pathways$displayName)
names(pathwayList) <- pathways$stId
# Query pathway data using pathway IDs
REACTOMEGeneSets <- sapply(names(pathwayList), function (pwId) {
  genesID <- event2Ids(event.id = pwId)
  geneSymbol <- genesID[["geneSymbol"]]
  geneSymbol
})
names(REACTOMEGeneSets) <- names(pathwayList)
# Retrieve all the selected pathways
REACTOMEPathways <- pathwayList[names(REACTOMEGeneSets)]
# Show the first 5 REACTOME pathways
REACTOMEGeneSets[1:5]
```

$`R-HSA-15869` 1. 'RRM1' 2. 'RR1' 3. 'RRM2' 4. 'RR2' 5. 'GLRX' 6. 'GRX' 7. 'DCTD' 8. 'RRM2B' 9. 'P53R2' 10. 'TXN' 11. 'TRDX' 12. 'TRX' 13. 'TRX1' 14. 'GUK1' 15. 'GMK' 16. 'GMPK' 17. 'NME1' 18. 'NDPKA' 19. 'NM23' 20. 'NME2' 21. 'NM23B' 22. 'CMPK1' 23. 'CMK' 24. 'CMPK' 25. 'UCK' 26. 'UMK' 27. 'UMPK' 28. 'AK1' 29. 'NME4' 30. 'NM23D' 31. 'AK8' 32. 'C9orf98' 33. 'AK7' 34. 'AK9' 35. 'AKD1' 36. 'AKD2' 37. 'C6orf199' 38. 'C6orf224' 39. 'AK5' 40. 'NME2P1' 41. 'NME3' 42. 'DTYMK' 43. 'CDC8' 44. 'TMPK' 45. 'TYMK' 46. 'DCTPP1' 47. 'XTP3TPA' 48. 'CDA03' 49. 'CTPS2' 50. 'TXNRD1' 51. 'GRIM12' 52. 'KDRF' 53. 'CTPS1' 54. 'CTPS' 55. 'AK6' 56. 'CINAP' 57. 'AD-004' 58. 'CGI-137' 59. 'AK4' 60. 'AK3' 61. 'AK3L1' 62. 'NUDT13' 63. 'TYMS' 64. 'TS' 65. 'OK/SW-cl.29' 66. 'DUT' 67. 'GSR' 68. 'GLUR' 69. 'GRD1' 70. 'AK2' 71. 'ADK2' 72. 'XDH' 73. 'XDHA' 74. 'ITPA' 75. 'C20orf37' 76. 'My049' 77. 'OK/SW-cl.9' 78. 'PNP' 79. 'NP' 80. 'NT5C1B' 81. 'AIRP' 82. 'FKSG85' 83. 'NT5C2' 84. 'NT5B' 85. 'NT5CP' 86. 'PNT5' 87. 'NT5C1A' 88. 'GDA' 89. 'KIAA1258' 90. 'NUDT15' 91. 'MTH2' 92. 'NUDT18' 93. 'MTH3' 94. 'NUDT1' 95. 'MTH1' 96. 'NUDT1' 97. 'MTH1' 98. 'NUDT1' 99. 'MTH1' 100. 'NUDT1' 101. 'MTH1' 102. 'NUDT9' 103. 'NUDT10' 104. 'PSEC0099' 105. 'UNQ3012/PRO9771' 106. 'NUDT16' 107. 'ADPRM' 108. 'C17orf48' 109. 'MDS006' 110. 'Nbla03831' 111. 'NUDT5' 112. 'NUDIX5' 113. 'HSPC115' 114. 'DNPH1' 115. 'C6orf108' 116. 'RCL' 117. 'NT5E' 118. 'NT5' 119. 'NTE' 120. 'NT5C' 121. 'DNT1' 122. 'UMPH2' 123. 'POMP' 124. 'NT5C3A' 125. 'NT5C3' 126. 'P5N1' 127. 'UMPH1' 128. 'HSPC233' 129. 'TYMP' 130. 'ECGF1' 131. 'UPP1' 132. 'UP' 133. 'UPP2' 134. 'NT5M' 135. 'DNT2' 136. 'DPYD' 137. 'DPYS' 138. 'UPB1' 139. 'BUP1' 140. 'AGXT2' 141. 'AGT2' 142. 'ENTPD1' 143. 'CD39' 144. 'ENTPD3' 145. 'CD39L3' 146. 'ENTPD7' 147. 'LALP1' 148. 'ENTPD6' 149. 'CD39L2' 150. 'IL6ST2' 151. 'ENTPD4' 152. 'KIAA0392' 153. 'LALP70' 154. 'LYSAL1' 155. 'ENTPD8' 156. 'UNQ2492/PRO5779' 157. 'ENTPD2' 158. 'CD39L1' 159. 'ENTPD5' 160. 'CD39L4' 161. 'PCPH' 162. 'SAMHD1' 163. 'MOP5' 164. 'DHODH' 165. 'CAD' 166. 'UMPS' 167. 'OK/SW-cl.21' 168. 'GMPS' 169. 'IMPDH2' 170. 'IMPD2' 171. 'IM-

PDH1' 172. 'IMPD1' 173. 'ATIC' 174. 'PURH' 175. 'OK/SW-cl.86' 176. 'ADSS' 177. 'ADSS2' 178. 'ADSSL1' 179. 'ADSS1' 180. 'ADSL' 181. 'AMPS' 182. 'PAICS' 183. 'ADE2' 184. 'AIRC' 185. 'PAIS' 186. 'GART' 187. 'PGFT' 188. 'PRGS' 189. 'PPAT' 190. 'GPAT' 191. 'PFAS' 192. 'KIAA0361' 193. 'ADA' 194. 'ADA1' 195. 'ADAL' 196. 'ADAL1' 197. 'APRT' 198. 'DCK' 199. 'HPRT1' 200. 'HPRT' 201. 'ADK' 202. 'GMPR2' 203. 'GMPR' 204. 'GMPR1' 205. 'DGUOK' 206. 'DGK' 207. 'AMPD1' 208. 'AMPD3' 209. 'AMPD2' 210. 'PUDP' 211. 'DXF68S1E' 212. 'FAM16AX' 213. 'GS1' 214. 'HDHD1' 215. 'HDHD1A' 216. 'TK1' 217. 'TK' 218. 'Thymidine kinase' 219. 'CDA' 220. 'CDD' 221. 'TK2' 222. 'UCK1' 223. 'URK1' 224. 'UCK2' 225. 'UMPK' 226. 'UCKL1' 227. 'URKL1' 228. 'F538'

$‘R-HSA-68616‘ 1. 'ORC1' 2. 'ORC1L' 3. 'PARC1' 4. 'H4C1' 5. 'H4/A' 6. 'H4FA' 7. 'HIST1H4A' 8. 'H4C2' 9. 'H4/I' 10. 'H4FI' 11. 'HIST1H4B' 12. 'H4C3' 13. 'H4/G' 14. 'H4FG' 15. 'HIST1H4C' 16. 'H4C4' 17. 'H4/B' 18. 'H4FB' 19. 'HIST1H4D' 20. 'H4C5' 21. 'H4/J' 22. 'H4FJ' 23. 'HIST1H4E' 24. 'H4C6' 25. 'H4/C' 26. 'H4FC' 27. 'HIST1H4F' 28. 'H4C8' 29. 'H4/H' 30. 'H4FH' 31. 'HIST1H4H' 32. 'H4C9' 33. 'H4/M' 34. 'H4FM' 35. 'HIST1H4I' 36. 'H4C11' 37. 'H4/E' 38. 'H4FE' 39. 'HIST1H4J' 40. 'H4C12' 41. 'H4/D' 42. 'H4FD' 43. 'HIST1H4K' 44. 'H4C13' 45. 'H4/K' 46. 'H4FK' 47. 'HIST1H4L' 48. 'H4C14' 49. 'H4/N' 50. 'H4F2' 51. 'H4FN' 52. 'HIST2H4' 53. 'HIST2H4A' 54. 'H4C15' 55. 'H4/O' 56. 'H4FO' 57. 'HIST2H4B' 58. 'H4C16' 59. 'H4-16' 60. 'HIST4H4' 61. 'H2BC14' 62. 'H2BFE' 63. 'HIST1H2BM' 64. 'H2BC1' 65. 'HIST1H2BA' 66. 'TSH2B' 67. 'H2BC5' 68. 'H2BFB' 69. 'HIRIP2' 70. 'HIST1H2BD' 71. 'H2BC9' 72. 'H2BFJ' 73. 'HIST1H2BH' 74. 'H2BC21' 75. 'H2BFQ' 76. 'HIST2H2BE' 77. 'H2BC17' 78. 'H2BFH' 79. 'H2BFN' 80. 'HIST1H2BO' 81. 'H2BC12L' 82. 'H2BFS' 83. 'H2BS1' 84. 'H2BC15' 85. 'H2BFD' 86. 'HIST1H2BN' 87. 'H2BC4' 88. 'H2BFL' 89. 'HIST1H2BC' 90. 'H2BC6' 91. 'H2BFH' 92. 'HIST1H2BE' 93. 'H2BC7' 94. 'H2BFG' 95. 'HIST1H2BF' 96. 'H2BC8' 97. 'H2BFA' 98. 'HIST1H2BG' 99. 'H2BC10' 100. 'H2BFK' 101. 'HIST1H2BI' 102. 'H2BC11' 103. 'H2BFR' 104. 'HIST1H2BJ' 105. 'H2BC3' 106. 'H2BFF' 107. 'HIST1H2BB' 108. 'H2BC12' 109. 'H2BFT' 110. 'HIRIP1' 111. 'HIST1H2BK' 112. 'H2BC13' 113. 'H2BFC' 114. 'HIST1H2BL' 115. 'H2BC26' 116. 'H2BU1' 117. 'HIST3H2BB' 118. 'H3-3A' 119. 'H3.3A' 120. 'H3F3' 121. 'H3F3A' 122. 'PP781' 123. 'H3-3B' 124. 'H3.3B' 125. 'H3F3B' 126. 'HIST1H3G' 127. 'HIST1H3A' 128. 'H3FA' 129. 'HIST1H3B' 130. 'H3FL' 131. 'HIST1H3C' 132. 'H3FC' 133. 'HIST1H3D' 134. 'H3FB' 135. 'HIST1H3E' 136. 'H3FD' 137. 'HIST1H3F' 138. 'H3FI' 139. 'H3FH' 140. 'HIST1H3H' 141. 'H3FK' 142. 'HIST1H3I' 143. 'H3FF' 144. 'HIST1H3J' 145. 'H3FJ' 146. 'H3C1' 147. 'H3C2' 148. 'H3C3' 149. 'H3FCHIST1H3C' 150. 'H3C4' 151. 'H3C6' 152. 'H3C7' 153. 'H3C8' 154. 'H3C10' 155. 'H3C11' 156. 'H3C12' 157. 'H3C15' 158. 'HIST2H3A' 159. 'H3C14' 160. 'H3F2' 161. 'H3FM' 162. 'HIST2H3C' 163. 'H3C13' 164. 'HIST2H3D' 165. 'H2AB1' 166. 'H2AFB1' 167. 'H2AZ2' 168. 'H2AFV' 169. 'H2AV' 170. 'H2AC7' 171. 'H2AFG' 172. 'HIST1H2AD' 173. 'H2AFX' 174. 'H2AX' 175. 'H2AJ' 176. 'H2AFJ' 177. 'H2AC20' 178. 'H2AFQ' 179. 'HIST2H2AC' 180. 'H2AC6' 181. 'H2AFL' 182. 'HIST1H2AC' 183. 'H2AC4' 184. 'H2AFM' 185. 'HIST1H2AB' 186. 'H2AC8' 187. 'H2AFA' 188. 'HIST1H2AE' 189. 'H2AC18' 190. 'H2AFO' 191. 'HIST2H2AA' 192. 'HIST2H2AA3' 193. 'H2AC19' 194. 'HIST2H2AA4' 195. 'H2AC14' 196. 'H2AFE' 197. 'HIST1H2AJ' 198. 'KPNA6' 199. 'IPOA7' 200. 'KPNA1' 201. 'RCH2' 202. 'KPNB1' 203. 'NTF97' 204. 'ORC6' 205. 'ORC6L' 206. 'ORC5' 207. 'ORC5L' 208. 'ORC3' 209. 'LATHEO' 210. 'ORC3L' 211. 'ORC2' 212. 'ORC2L' 213. 'ORC4' 214. 'ORC4L'

$‘R-HSA-68689‘ 1. 'ORC1' 2. 'ORC1L' 3. 'PARC1' 4. 'ORC4' 5. 'ORC4L' 6. 'ORC5' 7. 'ORC5L' 8. 'ORC3' 9. 'LATHEO' 10. 'ORC3L' 11. 'ORC2' 12. 'ORC2L' 13. 'ORC6' 14. 'ORC6L' 15. 'CDC6' 16. 'CDC18L' 17. 'MCM8' 18. 'C20orf154'

$‘R-HSA-68867‘ 1. ’ORC1’ 2. ’ORC1L’ 3. ’PARC1’ 4. ’ORC4’ 5. ’ORC4L’ 6. ’ORC5’ 7. ’ORC5L’ 8. ’ORC3’ 9. ’LATHEO’ 10. ’ORC3L’ 11. ’ORC2’ 12. ’ORC2L’ 13. ’ORC6’ 14. ’ORC6L’ 15. ’CDC6’ 16. ’CDC18L’ 17. ’MCM8’ 18. ’C20orf154’ 19. ’MCM4’ 20. ’CDC21’ 21. ’MCM6’ 22. ’MCM7’ 23. ’CDC47’ 24. ’MCM2’ 25. ’MCM2’ 26. ’BM28’ 27. ’CCNL1’ 28. ’CDCL1’ 29. ’KIAA0030’ 30. ’MCM3’ 31. ’MCM5’ 32. ’CDC46’ 33. ’CDT1’ 34. ’PSMA1’ 35. ’HC2’ 36. ’NU’ 37. ’PROS30’ 38. ’PSC2’ 39. ’PSMC2’ 40. ’MSS1’ 41. ’PSMB2’ 42. ’PSMB9’ 43. ’LMP2’ 44. ’PSMB6i’ 45. ’RING12’ 46. ’PSMD14’ 47. ’POH1’ 48. ’PSMD3’ 49. ’PSMB7’ 50. ’Z’ 51. ’PSMD4’ 52. ’MCB1’ 53. ’PSMA4’ 54. ’HC9’ 55. ’PSC9’ 56. ’PSMD11’ 57. ’PSMD13’ 58. ’PSMD9’ 59. ’PSME1’ 60. ’IFI5111’ 61. ’PSMA3’ 62. ’HC8’ 63. ’PSC8’ 64. ’PSMD10’ 65. ’SEM1’ 66. ’DSS1’ 67. ’SHFDG1’ 68. ’SHFM1’ 69. ’C7orf76’ 70. ’PSMD6’ 71. ’KIAA0107’ 72. ’PFAAP4’ 73. ’PSMC6’ 74. ’SUG2’ 75. ’PSMD8’ 76. ’PSMC3’ 77. ’TBP1’ 78. ’PSMC5’ 79. ’SUG1’ 80. ’PSMC4’ 81. ’MIP224’ 82. ’TBP7’ 83. ’PSMA6’ 84. ’PROS27’ 85. ’PSMD7’ 86. ’MOV34L’ 87. ’PSMF1’ 88. ’PSMD2’ 89. ’TRAP2’ 90. ’PSME3’ 91. ’PSMB10’ 92. ’LMP10’ 93. ’MECL1’ 94. ’PSMD1’ 95. ’PSMA7’ 96. ’HSPC’ 97. ’PSME2’ 98. ’PSMA2’ 99. ’HC3’ 100. ’PSC3’ 101. ’PSMB5’ 102. ’LMPX’ 103. ’MB1’ 104. ’X’ 105. ’PSMB1’ 106. ’PSC5’ 107. ’PSMD5’ 108. ’KIAA0072’ 109. ’PSMB3’ 110. ’PSMA5’ 111. ’PSMB4’ 112. ’PROS26’ 113. ’PSMB6’ 114. ’LMPY’ 115. ’Y’ 116. ’PSMB8’ 117. ’LMP7’ 118. ’PSMB5i’ 119. ’RING10’ 120. ’Y2’ 121. ’PSMD12’ 122. ’PSMC1’ 123. ’GMNN’ 124. ’UBC’ 125. ’UBB’ 126. ’UBA52’ 127. ’UBCEP2’ 128. ’RPS27A’ 129. ’UBA80’ 130. ’UBCEP1’ 131. ’H4C1’ 132. ’H4/A’ 133. ’H4FA’ 134. ’HIST1H4A’ 135. ’H4C2’ 136. ’H4/I’ 137. ’H4FI’ 138. ’HIST1H4B’ 139. ’H4C3’ 140. ’H4/G’ 141. ’H4FG’ 142. ’HIST1H4C’ 143. ’H4C4’ 144. ’H4/B’ 145. ’H4FB’ 146. ’HIST1H4D’ 147. ’H4C5’ 148. ’H4/J’ 149. ’H4FJ’ 150. ’HIST1H4E’ 151. ’H4C6’ 152. ’H4/C’ 153. ’H4FC’ 154. ’HIST1H4F’ 155. ’H4C8’ 156. ’H4/H’ 157. ’H4FH’ 158. ’HIST1H4H’ 159. ’H4C9’ 160. ’H4/M’ 161. ’H4FM’ 162. ’HIST1H4I’ 163. ’H4C11’ 164. ’H4/E’ 165. ’H4FE’ 166. ’HIST1H4J’ 167. ’H4C12’ 168. ’H4/D’ 169. ’H4FD’ 170. ’HIST1H4K’ 171. ’H4C13’ 172. ’H4/K’ 173. ’H4FK’ 174. ’HIST1H4L’ 175. ’H4C14’ 176. ’H4/N’ 177. ’H4F2’ 178. ’H4FN’ 179. ’HIST2H4’ 180. ’HIST2H4A’ 181. ’H4C15’ 182. ’H4/O’ 183. ’H4FO’ 184. ’HIST2H4B’ 185. ’H4C16’ 186. ’H4-16’ 187. ’HIST4H4’ 188. ’H2BC14’ 189. ’H2BFE’ 190. ’HIST1H2BM’ 191. ’H2BC1’ 192. ’HIST1H2BA’ 193. ’TSH2B’ 194. ’H2BC5’ 195. ’H2BFB’ 196. ’HIRIP2’ 197. ’HIST1H2BD’ 198. ’H2BC9’ 199. ’H2BFJ’ 200. ’HIST1H2BH’ 201. ’H2BC21’ 202. ’H2BFQ’ 203. ’HIST2H2BE’ 204. ’H2BC17’ 205. ’H2BFH’ 206. ’H2BFN’ 207. ’HIST1H2BO’ 208. ’H2BC12L’ 209. ’H2BFS’ 210. ’H2BS1’ 211. ’H2BC15’ 212. ’H2BFD’ 213. ’HIST1H2BN’ 214. ’H2BC4’ 215. ’H2BFL’ 216. ’HIST1H2BC’ 217. ’H2BC6’ 218. ’H2BFH’ 219. ’HIST1H2BE’ 220. ’H2BC7’ 221. ’H2BFG’ 222. ’HIST1H2BF’ 223. ’H2BC8’ 224. ’H2BFA’ 225. ’HIST1H2BG’ 226. ’H2BC10’ 227. ’H2BFK’ 228. ’HIST1H2BI’ 229. ’H2BC11’ 230. ’H2BFR’ 231. ’HIST1H2BJ’ 232. ’H2BC3’ 233. ’H2BFF’ 234. ’HIST1H2BB’ 235. ’H2BC12’ 236. ’H2BFT’ 237. ’HIRIP1’ 238. ’HIST1H2BK’ 239. ’H2BC13’ 240. ’H2BFC’ 241. ’HIST1H2BL’ 242. ’H2BC26’ 243. ’H2BU1’ 244. ’HIST3H2BB’ 245. ’H3-3A’ 246. ’H3.3A’ 247. ’H3F3’ 248. ’H3F3A’ 249. ’PP781’ 250. ’H3-3B’ 251. ’H3.3B’ 252. ’H3F3B’ 253. ’HIST1H3G’ 254. ’HIST1H3A’ 255. ’H3FA’ 256. ’HIST1H3B’ 257. ’H3FL’ 258. ’HIST1H3C’ 259. ’H3FC’ 260. ’HIST1H3D’ 261. ’H3FB’ 262. ’HIST1H3E’ 263. ’H3FD’ 264. ’HIST1H3F’ 265. ’H3FI’ 266. ’H3FH’ 267. ’HIST1H3H’ 268. ’H3FK’ 269. ’HIST1H3I’ 270. ’H3FF’ 271. ’HIST1H3J’ 272. ’H3FJ’ 273. ’H3C1’ 274. ’H3C2’ 275. ’H3C3’ 276. ’H3FCHIST1H3C’ 277. ’H3C4’ 278. ’H3C6’ 279. ’H3C7’ 280. ’H3C8’ 281. ’H3C10’ 282. ’H3C11’ 283. ’H3C12’ 284. ’H3C15’ 285. ’HIST2H3A’ 286. ’H3C14’ 287. ’H3F2’ 288. ’H3FM’ 289. ’HIST2H3C’ 290. ’H3C13’ 291. ’HIST2H3D’ 292. ’H2AB1’ 293. ’H2AFB1’ 294. ’H2AZ2’ 295. ’H2AFV’ 296. ’H2AV’ 297. ’H2AC7’ 298. ’H2AFG’ 299. ’HIST1H2AD’ 300. ’H2AFX’ 301. ’H2AX’ 302. ’H2AJ’

303. 'H2AFJ' 304. 'H2AC20' 305. 'H2AFQ' 306. 'HIST2H2AC' 307. 'H2AC6' 308. 'H2AFL' 309. 'HIST1H2AC' 310. 'H2AC4' 311. 'H2AFM' 312. 'HIST1H2AB' 313. 'H2AC8' 314. 'H2AFA' 315. 'HIST1H2AE' 316. 'H2AC18' 317. 'H2AFO' 318. 'HIST2H2AA' 319. 'HIST2H2AA3' 320. 'H2AC19' 321. 'HIST2H2AA4' 322. 'H2AC14' 323. 'H2AFE' 324. 'HIST1H2AJ' 325. 'KPNA6' 326. 'IPOA7' 327. 'KPNA1' 328. 'RCH2' 329. 'KPNB1' 330. 'NTF97' 331. 'FZR1' 332. 'CDH1' 333. 'FYR' 334. 'FZR' 335. 'KIAA1242' 336. 'ANAPC4' 337. 'APC4' 338. 'CDC16' 339. 'ANAPC6' 340. 'CDC26' 341. 'ANAPC12' 342. 'C9orf17' 343. 'ANAPC1' 344. 'TSG24' 345. 'ANAPC5' 346. 'APC5' 347. 'UBE2E1' 348. 'UBCH6' 349. 'UBE2C' 350. 'UBCH10' 351. 'ANAPC11' 352. 'HSPC214' 353. 'UBE2S' 354. 'E2EPF' 355. 'OK/SW-cl.73' 356. 'CDC23' 357. 'ANAPC8' 358. 'ANAPC16' 359. 'C10orf104' 360. 'CENP-27' 361. 'UBE2D1' 362. 'SFT' 363. 'UBC5A' 364. 'UBCH5' 365. 'UBCH5A' 366. 'ANAPC15' 367. 'C11orf51' 368. 'HSPC020' 369. 'CDC27' 370. 'ANAPC3' 371. 'D0S1430E' 372. 'D17S978E' 373. 'ANAPC10' 374. 'APC10' 375. 'ANAPC7' 376. 'APC7' 377. 'ANAPC2' 378. 'APC2' 379. 'KIAA1406'

$'R-HSA-68875' 1. 'PLK1' 2. 'PLK' 3. 'GOLGA2' 4. 'GORASP1' 5. 'GOLPH5' 6. 'GRASP65' 7. 'RAB1B' 8. 'RAB1A' 9. 'RAB1' 10. 'MAPK3' 11. 'ERK1' 12. 'PRKM3' 13. 'MAPK1' 14. 'ERK2' 15. 'PRKM1' 16. 'PRKM2' 17. 'BLZF1' 18. 'JEM1' 19. 'GORASP2' 20. 'GOLPH6' 21. 'RAB2A' 22. 'RAB2' 23. 'CDK1' 24. 'CDC2' 25. 'CDC28A' 26. 'CDKN1' 27. 'P34CDC2' 28. 'CCNB1' 29. 'CCNB' 30. 'CCNB2' 31. 'USO1' 32. 'VDP' 33. 'MASTL' 34. 'GW' 35. 'GWL' 36. 'THC2' 37. 'ENSA' 38. 'ARPP19' 39. 'PPP2R2D' 40. 'KIAA1541' 41. 'PPP2CB' 42. 'PPP2CA' 43. 'PPP2R1A' 44. 'PPP2R1B' 45. 'NEK9' 46. 'KIAA1995' 47. 'NEK8' 48. 'NERCC' 49. 'NEK7' 50. 'NEK6' 51. 'LPIN2' 52. 'KIAA0249' 53. 'LPIN3' 54. 'LIPN3L' 55. 'LPIN1' 56. 'KIAA0188' 57. 'PRKCB' 58. 'PKC-beta' 59. 'PKCB' 60. 'PRKCB1' 61. 'PRKCA' 62. 'PKCA' 63. 'PRKACA' 64. 'LMNB1' 65. 'LMN2' 66. 'LMNB' 67. 'LMNA' 68. 'LMN1' 69. 'LMNA' 70. 'LMN1' 71. 'TMPO' 72. 'LAP2' 73. 'LEMD3' 74. 'MAN1' 75. 'LEMD2' 76. 'EMD' 77. 'EDMD' 78. 'STA' 79. 'CNEP1R1' 80. 'C16orf69' 81. 'TMEM188' 82. 'CTDNEP1' 83. 'DULLARD' 84. 'VRK2' 85. 'VRK1' 86. 'BANF1' 87. 'BAF' 88. 'BCRG1' 89. 'NUP42' 90. 'CG1' 91. 'NUPL2' 92. 'RANBP2' 93. 'NUP358' 94. 'AAAS' 95. 'ADRACALA' 96. 'GL003' 97. 'NUP50' 98. 'NPAP60L' 99. 'PRO1146' 100. 'NUP62' 101. 'NUP58' 102. 'KIAA0410' 103. 'NUPL1' 104. 'NUP58' 105. 'KIAA0410' 106. 'NUPL1' 107. 'NUP54' 108. 'RAE1' 109. 'MRNP41' 110. 'SEH1L' 111. 'SEC13L' 112. 'SEH1' 113. 'NUP35' 114. 'MP44' 115. 'NUP53' 116. 'NUP93' 117. 'KIAA0095' 118. 'NUP155' 119. 'KIAA0791' 120. 'NUP205' 121. 'C7orf14' 122. 'KIAA0225' 123. 'NUP98' 124. 'ADAR2' 125. 'NUP98' 126. 'ADAR2' 127. 'POM121' 128. 'KIAA0618' 129. 'NUP121' 130. 'POM121A' 131. 'POM121C' 132. 'NUP210' 133. 'KIAA0906' 134. 'PSEC0245' 135. 'NDC1' 136. 'TMEM48' 137. 'NUP214' 138. 'CAIN' 139. 'CAN' 140. 'KIAA0023' 141. 'NUP188' 142. 'KIAA0169' 143. 'NUP88' 144. 'NUP153' 145. 'TPR' 146. 'NUP98' 147. 'ADAR2' 148. 'SEH1L' 149. 'SEC13L' 150. 'SEH1' 151. 'NUP107' 152. 'NUP43' 153. 'NUP160' 154. 'KIAA0197' 155. 'NUP120' 156. 'NUP37' 157. 'NUP85' 158. 'NUP75' 159. 'PCNT1' 160. 'NUP133' 161. 'SEC13' 162. 'D3S1231E' 163. 'SEC13A' 164. 'SEC13L1' 165. 'SEC13R' 166. 'NUMA1' 167. 'NMP22' 168. 'NUMA' 169. 'PHF8' 170. 'KIAA1111' 171. 'ZNF422' 172. 'PHF8' 173. 'KIAA1111' 174. 'ZNF422' 175. 'PHF8' 176. 'KIAA1111' 177. 'ZNF422' 178. 'H4C1' 179. 'H4/A' 180. 'H4FA' 181. 'HIST1H4A' 182. 'H4C2' 183. 'H4/I' 184. 'H4FI' 185. 'HIST1H4B' 186. 'H4C3' 187. 'H4/G' 188. 'H4FG' 189. 'HIST1H4C' 190. 'H4C4' 191. 'H4/B' 192. 'H4FB' 193. 'HIST1H4D' 194. 'H4C5' 195. 'H4/J' 196. 'H4FJ' 197. 'HIST1H4E' 198. 'H4C6' 199. 'H4/C' 200. 'H4FC' 201. 'HIST1H4F' 202. 'H4C8' 203. 'H4/H' 204. 'H4FH'

205. 'HIST1H4H' 206. 'H4C9' 207. 'H4/M' 208. 'H4FM' 209. 'HIST1H4I' 210. 'H4C11' 211. 'H4/E' 212. 'H4FE' 213. 'HIST1H4J' 214. 'H4C12' 215. 'H4/D' 216. 'H4FD' 217. 'HIST1H4K' 218. 'H4C13' 219. 'H4/K' 220. 'H4FK' 221. 'HIST1H4L' 222. 'H4C14' 223. 'H4/N' 224. 'H4F2' 225. 'H4FN' 226. 'HIST2H4' 227. 'HIST2H4A' 228. 'H4C15' 229. 'H4/O' 230. 'H4FO' 231. 'HIST2H4B' 232. 'H4C16' 233. 'H4-16' 234. 'HIST4H4' 235. 'H2BC14' 236. 'H2BFE' 237. 'HIST1H2BM' 238. 'H2BC1' 239. 'HIST1H2BA' 240. 'TSH2B' 241. 'H2BC5' 242. 'H2BFB' 243. 'HIRIP2' 244. 'HIST1H2BD' 245. 'H2BC9' 246. 'H2BFJ' 247. 'HIST1H2BH' 248. 'H2BC21' 249. 'H2BFQ' 250. 'HIST2H2BE' 251. 'H2BC17' 252. 'H2BFH' 253. 'H2BFN' 254. 'HIST1H2BO' 255. 'H2BC12L' 256. 'H2BFS' 257. 'H2BS1' 258. 'H2BC15' 259. 'H2BFD' 260. 'HIST1H2BN' 261. 'H2BC4' 262. 'H2BFL' 263. 'HIST1H2BC' 264. 'H2BC6' 265. 'H2BFH' 266. 'HIST1H2BE' 267. 'H2BC7' 268. 'H2BFG' 269. 'HIST1H2BF' 270. 'H2BC8' 271. 'H2BFA' 272. 'HIST1H2BG' 273. 'H2BC10' 274. 'H2BFK' 275. 'HIST1H2BI' 276. 'H2BC11' 277. 'H2BFR' 278. 'HIST1H2BJ' 279. 'H2BC3' 280. 'H2BFF' 281. 'HIST1H2BB' 282. 'H2BC12' 283. 'H2BFT' 284. 'HIRIP1' 285. 'HIST1H2BK' 286. 'H2BC13' 287. 'H2BFC' 288. 'HIST1H2BL' 289. 'H2BC26' 290. 'H2BU1' 291. 'HIST3H2BB' 292. 'H3-4' 293. 'H3FT' 294. 'HIST3H3' 295. 'HIST1H3G' 296. 'HIST1H3A' 297. 'H3FA' 298. 'HIST1H3B' 299. 'H3FL' 300. 'HIST1H3C' 301. 'H3FC' 302. 'HIST1H3D' 303. 'H3FB' 304. 'HIST1H3E' 305. 'H3FD' 306. 'HIST1H3F' 307. 'H3FI' 308. 'H3FH' 309. 'HIST1H3H' 310. 'H3FK' 311. 'HIST1H3I' 312. 'H3FF' 313. 'HIST1H3J' 314. 'H3FJ' 315. 'H3C1' 316. 'H3C2' 317. 'H3C3' 318. 'H3FCHIST1H3C' 319. 'H3C4' 320. 'H3C6' 321. 'H3C7' 322. 'H3C8' 323. 'H3C10' 324. 'H3C11' 325. 'H3C12' 326. 'H3-3A' 327. 'H3.3A' 328. 'H3F3' 329. 'H3F3A' 330. 'PP781' 331. 'H3-3B' 332. 'H3.3B' 333. 'H3F3B' 334. 'H3C15' 335. 'HIST2H3A' 336. 'H3C14' 337. 'H3F2' 338. 'H3FM' 339. 'HIST2H3C' 340. 'H3C13' 341. 'HIST2H3D' 342. 'H2AB1' 343. 'H2AFB1' 344. 'H2AZ2' 345. 'H2AFV' 346. 'H2AV' 347. 'H2AC7' 348. 'H2AFG' 349. 'HIST1H2AD' 350. 'H2AFX' 351. 'H2AX' 352. 'H2AJ' 353. 'H2AFJ' 354. 'H2AC20' 355. 'H2AFQ' 356. 'HIST2H2AC' 357. 'H2AC6' 358. 'H2AFL' 359. 'HIST1H2AC' 360. 'H2AC4' 361. 'H2AFM' 362. 'HIST1H2AB' 363. 'H2AC8' 364. 'H2AFA' 365. 'HIST1H2AE' 366. 'H2AC18' 367. 'H2AFO' 368. 'HIST2H2AA' 369. 'HIST2H2AA3' 370. 'H2AC19' 371. 'HIST2H2AA4' 372. 'H2AC14' 373. 'H2AFE' 374. 'HIST1H2AJ' 375. 'MCPH1' 376. 'SET' 377. 'NCAPD3' 378. 'CAPD3' 379. 'KIAA0056' 380. 'SMC2' 381. 'CAPE' 382. 'SMC2L1' 383. 'PRO0324' 384. 'SMC4' 385. 'CAPC' 386. 'SMC4L1' 387. 'NCAPH2' 388. 'CAPH2' 389. 'NCAPG2' 390. 'LUZP5' 391. 'RB1' 392. 'KMT5A' 393. 'PRSET7' 394. 'SET07' 395. 'SET8' 396. 'SETD8'

4 Submodule 04: Pathway Analysis

```
# Install the fgsea package
suppressMessages({
    if (!require("BiocManager", quietly = TRUE)) {
        install.packages("BiocManager")
    }
    suppressWarnings(BiocManager::install("fgsea", update = F))

})
# Loading the package
```

```
suppressPackageStartupMessages({
    library("fgsea")
})
```

```
#' @description This function performs gene set analysis using fgsea (fast gene␣
↪set enrichment analysis).
#'
#' @param DE_data A dataframe containing the DE analysis result.
#' @param genesets A list of gene sets to be analyzed, ex. KEGG genesets.
#' @param statCol A character string specifying the name of a statistic column␣
↪in DE_data to be used in the EA analysis.
#' @param nperms An integer specifying the number of permutations for fgsea.
#' @param ncores An integer specifying the number cpu cores for parallel␣
↪processing.
#' @return A list containing a dataframe of gene set analysis results and a␣
↪statistic vector

runFgsea <- function(DE_data, genesets, statCol = "statistic", nperms = 200,␣
↪ncores = 0) {

    if (is.null(DE_data) |
        dim(DE_data)[1] == 0 |
        dim(DE_data)[2] == 0) {
        stop("No differential analysis data is in input data.")
    }

    DE_data <- DE_data[!is.na(DE_data[[statCol]]),]

    statistic <- unlist(DE_data[[statCol]])
    statistic <- as.vector(statistic)
    names(statistic) <- rownames(DE_data)
    set.seed(1)
    suppressWarnings(fgsea_res <- fgsea::fgsea(pathways = genesets, stats =␣
↪statistic, nperm = nperms, nproc = ncores))

    fgsea_res <- fgsea_res[!is.na(fgsea_res[["pval"]]), ]

    fgsea_res[,c("pathway", "pval", "padj", "ES", "NES")]
}
```

4.1 Running FGSEA on Microarray Dataset: GSE5281

```
gse5281KEGGFGSEA <- runFgsea(DE_data = gse5281LimmaRes, genesets = KEGGGeneSets,␣
↪nperms = 1000)
head(gse5281KEGGFGSEA, 3)
```

A data.table: 3 × 5

pathway <chr>	pval <dbl>	padj <dbl>	ES <dbl>	NES <dbl>
hsa00010	0.002478315	0.01900196	-0.5076268	-1.689699
hsa00020	0.001315789	0.01367188	-0.6974271	-2.034788
hsa00030	0.010638298	0.05555556	-0.5588607	-1.638633

4.2 Running FGSEA on RNA-Seq Dataset: GSE153873

```
gse153873KEGGFGSEA <- runFgsea(DE_data = gse153873DESeq2Res, genesets =␣
↪KEGGGeneSets, nperms = 1000)
head(gse153873KEGGFGSEA, 3)
```

A data.table: 3 × 5

pathway <chr>	pval <dbl>	padj <dbl>	ES <dbl>	NES <dbl>
hsa00010	0.001706485	0.0121259	-0.5694929	-2.281996
hsa00020	0.001841621	0.0121259	-0.7346406	-2.466437
hsa00030	0.001879699	0.0121259	-0.6423658	-2.159681

5 Submodule 05: Meta-analysis

```
# Install and import packages
install.packages("meta")
suppressWarnings(if (!require("ggnewscale")) install.packages("ggnewscale"))
suppressPackageStartupMessages({library(ggnewscale)})
```

```
The downloaded binary packages are in
/var/folders/lz/5chptxdx2yg5vvszdt29pt400000gn/T//RtmpmTTyNe/downloaded_packages
```

5.1 Perform Meta-analysis

```
# Combine KEGG FGSEA results from two datasets (GSE5281 and GSE153873)
keggFGSEAResults <- rbind(
  gse5281KEGGFGSEA %>% mutate(analysis = "GSE5281"),
  gse153873KEGGFGSEA %>% mutate(analysis = "GSE153873")
)

# Perform meta-analysis on KEGG FGSEA results for each pathway
metaResult <- keggFGSEAResults %>%
  group_by(pathway) %>%
  group_split() %>%
  lapply(function(df) {
    # Adjust p-values to avoid zero and one values
    df$padj[df$padj == 0] <- 1e-10
    df$padj[df$padj == 1] <- 1 - 1e-10
```

```r
    # Perform meta-analysis using the 'metagen' function
    res <- try({
      suppressWarnings({
        meta::metagen(
          TE = df$NES,
          pval = df$padj,
          sm = "SMD",
          method.tau = "REML",
          hakn = TRUE,
          warn. = FALSE
        )
      })
    })

    # Check for errors during meta-analysis and return NULL if encountered
    if ("try-error" %in% class(res)) {
      return(NULL)
    }

    # Create a data frame with meta-analysis results for each pathway
    data.frame(
      pathway = df$pathway[1],
      NES.combined = res$TE.fixed,
      NES.combined.sd = res$seTE.fixed,
      pval.combined = res$pval.fixed,
      stringsAsFactors = FALSE
    )
  }) %>%
  do.call(what = rbind)

# Adjust combined p-values using the Benjamini-Hochberg method
metaResult$pval.combined.adj <- p.adjust(metaResult$pval.combined, method = "BH")
# Show some first rows of the meta-result
head(metaResult, 3)
```

A data.frame: 3 × 5

	pathway <chr>	NES.combined <dbl>	NES.combined.sd <dbl>	pval.combined <dbl>	pval.combined.adj <dbl>
1	hsa00010	-1.917976	0.5647661	0.0006836508	0.005882995
2	hsa00020	-2.213178	0.6320963	0.0004629409	0.005259463
3	hsa00030	-1.897623	0.6069948	0.0017704607	0.014120503

5.2 Visualization of Analysis Result

5.2.1 Forest Plot

```
#' This function creates a forest plot to visualize pathway analysis results.
#'
#' @param pathways A vector of pathway names.
#' @param NES A vector of Normalized Enrichment Scores (NES) for each pathway.
#' @param padj A vector of adjusted p-values for each pathway.
#'
#' @return A ggplot object representing the forest plot.
plotForest <- function(pathways, NES, padj) {
  # Calculate standard deviation for error bars, limiting to a maximum of 1.5
  sd <- abs(NES / qnorm(padj))
  sd[sd > 1.5] <- 1.5

  # Create a ggplot object for the forest plot
  ggplot() + theme_minimal() +
    theme(
      panel.border = element_rect(colour = "black", fill = NA, linewidth=1),
      axis.text.y = element_blank()
    ) +
    # Plot points for NES with red color
    geom_point(aes(x = NES, y = pathways), size = 2, color = "red") +
    # Add error bars using standard deviation
    geom_errorbarh(aes(xmin = pmax(NES - sd * 2, -3), xmax = pmin(NES + sd * 2,␣
↪3), y = pathways), height = .1) +
    # Add vertical dashed lines at -1, 0, and 1
    geom_vline(xintercept = c(-1, 0, 1), colour = c("#FA8072", "black",␣
↪"#FA8072"), linetype = "longdash") +
    # Set x-axis limits
    xlim(-3, 3) +
    # Remove y-axis labels
    labs(y = NULL)
}

# Select the top pathways based on the combined adjusted p-value
topPathway <- metaResult %>% arrange(pval.combined.adj) %>% head(20) %>%␣
↪pull(pathway)
# Create plot data for the result of each dataset and the meta-result
gse5281PlotDat <- gse5281KEGGFGSEA[match(topPathway, gse5281KEGGFGSEA$pathway), ]
gse153873PlotDat <- gse153873KEGGFGSEA[match(topPathway,␣
↪gse153873KEGGFGSEA$pathway), ]
metaPlotDat <- metaResult[match(topPathway, metaResult$pathway), ]

# Create forest plots for the result of each dataset and the meta-result
p1 <- plotForest(gse5281PlotDat$pathway, gse5281PlotDat$NES, gse5281PlotDat$padj)
```

```r
p2 <- plotForest(gse153873PlotDat$pathway, gse153873PlotDat$NES,␣
 ↪gse153873PlotDat$padj)
p3 <- plotForest(metaPlotDat$pathway, metaPlotDat$NES.combined, metaPlotDat$pval.
 ↪combined.adj)
```

```r
options(repr.plot.width = 8, repr.plot.height = 6)

# Combine the three plots and show
plt <- gridExtra::grid.arrange(
    p1 + ggtitle("GSE5281") +
        scale_y_discrete(labels = gsub(" - Homo sapiens \\(human\\)", "",␣
 ↪KEGGPathways[topPathway])) +
        theme(axis.text.y = element_text(hjust = 1)),
    p2 + ggtitle("GSE153873"),
    p3 + ggtitle("Meta-analysis"),
    ncol = 3,
    widths = c(3.2,1,1)
)
plt
```

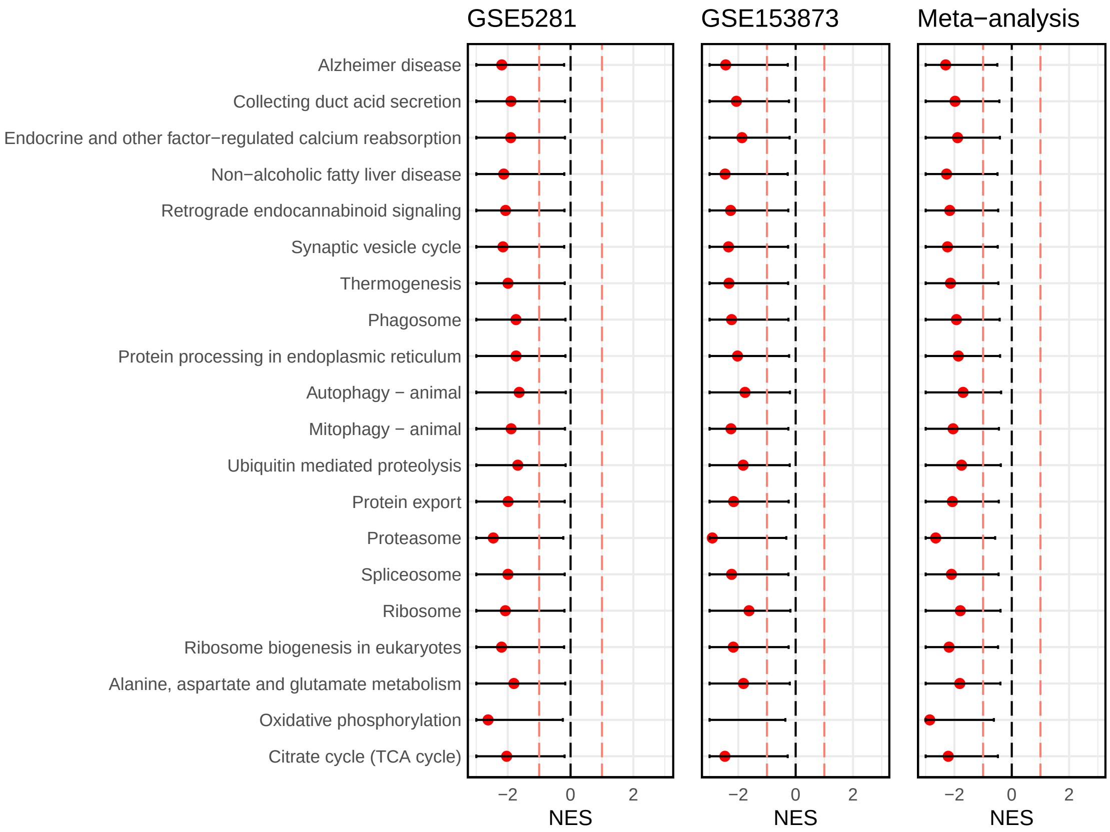

5.2.2 Pathway Venn Diagram

```r
#' @ description This function generates a Venn diagram to visualize the overlap␣
↪of significant pathways
#'                  between multiple sets of results.
#'
#' @param aResults A list containing multiple sets of pathway analysis results.
#'                  Each element in the list should be a data frame containing␣
↪pathway analysis results..
#' @param pThreshold The significance threshold for filtering differentially␣
↪expressed genes (default: 0.05).
#' @param useFDR A logical value indicating whether to use normal p-value or FDR␣
↪p-value to filter out the significant pathways
#'
#' @return A Venn diagram displaying the overlap of differentially expressed␣
↪genes or features among the specified result sets.

plotVenn <- function(aResults, pThreshold = 0.05, useFDR = TRUE) {
    if (length(aResults) < 2) {
        stop("The number of DE results must be at least 2.")
    }

    for (Res in aResults) {
        if (useFDR && !("padj" %in% colnames(Res))) {
            stop("The F DR adjusted p-value column is not in the results data␣
↪frame.")
        } else {
            if (!("pval" %in% colnames(Res))) {
                stop("The pval column is not in the results data frame.")
            }
        }

    }

    plotDat <- lapply(aResults, function(Res) {
            filtered_df <- filter(data.frame(Res),
                (
                    if (useFDR) {
                        .data$padj < pThreshold
                    } else {
                        .data$pval < pThreshold
                    }
                )
            )
            filtered_df[["pathway"]]
    })
```

```
    if (is.null(names(plotDat))) {
        names(plotDat) <- paste0("Dataset ", seq_along(plotDat))
    }
    pR <- ggvenn::ggvenn(plotDat,
           fill_color = c(
               "#316b9d",
               # "#fce397",
               # "#99cc83",
               "#f77a65",
               "#a6a1d0",
               "#fea9c4",
               "#74e7bc",
               "#febb73",
               "#1db4db",
               "#ffc5a6",
               "#b6c9fa",
               "#ee5437"),
           stroke_size = 0.5,
           set_name_size = 4,
           fill_alpha = 0.75,

    )

    pR
}
```

```
# Create input data for the plotVenn function
PAResults <- list("GSE5281" = gse5281KEGGFGSEA, "GSE153873" = gse153873KEGGFGSEA)
# Create the Venn diagram
pwVenn <- plotVenn(PAResults, useFDR = FALSE)
pwVenn
```

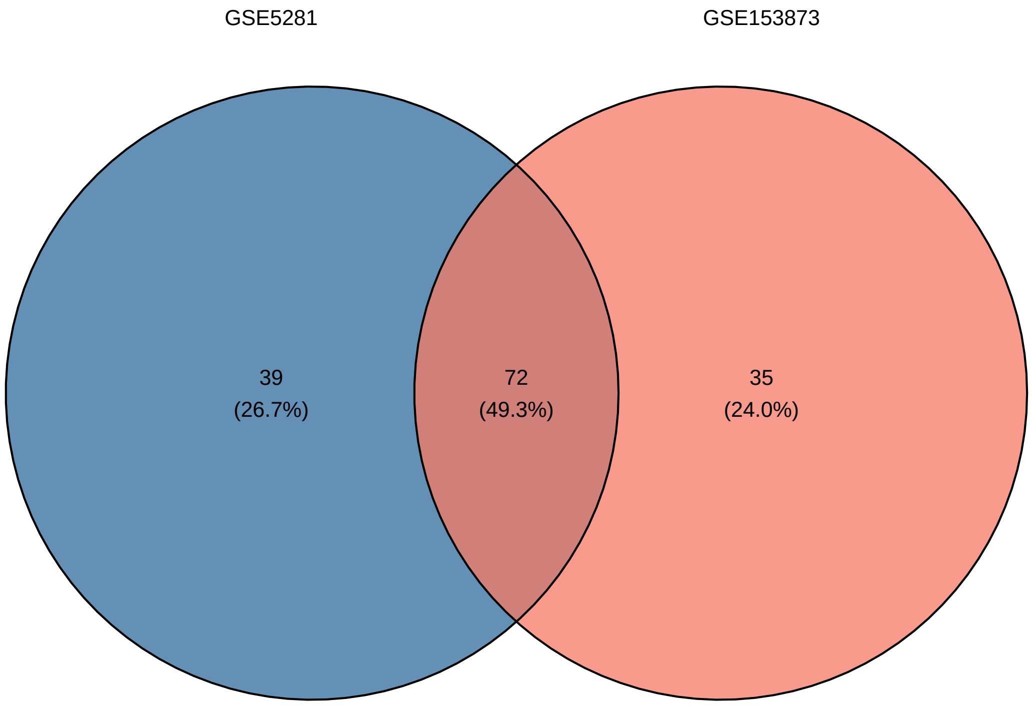

5.2.3 Pathway Heatmap Plot

```r
#' @description This function generates a heatmap visualizing pathway analysis␣
↪results.
#'
#' @param resultsList List of pathway analysis results.
#' @param yAxis Column to be used for the y-axis of the heatmap (default is␣
↪"pathway").
#' @param negLog10pValueLims Limits for the negative logarithm base 10 of␣
↪p-values (default is c(0, 5)).
#' @param useFDR Logical, whether to use False Discovery Rate (FDR) for p-values␣
↪(default is TRUE).
#' @param selectedPathways Vector of selected pathway IDs to include in the plot␣
↪(default is NULL).
#'
#' @return The function returns the heatmap plot.

plotPathwayHeatmap <- function(resultsList, yAxis = "pathway",␣
↪negLog10pValueLims = c(0, 5), useFDR = TRUE, selectedPathways = NULL) {

    if (!is.null(selectedPathways)) {
        resultsList <- lapply(resultsList, function(df) {
```

```r
            # Filter rows based on selectedPathways
            df <- df[df$pathway %in% selectedPathways, ]
            # Get the indices of selectedPathways in the original order
            indices <- match(df$pathway, selectedPathways)
            # Reorder the DataFrame based on the indices
            df <- df[order(indices), ]
            # Create a new column "name" with the names of selectedPathways
            df$name <- names(selectedPathways)
            df
        })
    }

    studyIDs <- names(resultsList)

    if (any(sapply(studyIDs, is.null))) {
        stop("The names of the input list should be specified.")
    }

    checkNS <- lapply(resultsList, function(data) c("NES") %in% colnames(data))

    resultsList <- lapply(seq_along(checkNS), function(i) {
      if (!checkNS[[i]]) {
        df <- resultsList[[i]]
        df$NES <- rep(.Machine$double.eps, nrow(df))
        resultsList[[i]] <- df
      } else {
        resultsList[[i]]
      }
    })

    cols_list <- lapply(resultsList, function(data) colnames(data))

    if (!all(sapply(cols_list, function(x) c("pathway", "NES", "pval") %in% x))) 
↪{
        stop("All dataframes in the input list must have 'pathway', 'NES', and 
↪'pval' columns.")
    }

    rows_list <- lapply(resultsList, function(data) as.
↪vector(unlist(data$pathway)))

    if (!all(lengths(rows_list) == length(rows_list[[1]]))) {
        stop("All dataframes in the input list must have the same number of rows.
↪")
    }

    initial_names <- sort(as.vector(unlist(resultsList[[1]]$pathway)))
```

```r
  if (!all(sapply(rows_list, function(x) all.equal(sort(x), initial_names)))) {
      stop("All dataframes in the input list must have the same set of␣
↪pathways.")
  }

  plotData <- lapply(1:length(resultsList), function(i) {
      data <- resultsList[[i]]
      data <- data[, c("pathway", "name", "NES", "pval")]
      data$dataset <- studyIDs[i]
      data$Direction <- ifelse(data$NES <= 0, "Negative", "Positive")
      data$abs.NES <- abs(as.vector(data$NES))
      as.data.frame(data)
  })

  plotData <- do.call(plotData, what = rbind)

  plotData$Direction <- factor(plotData$Direction, levels = c("Positive",␣
↪"Negative"))


  plotData$logP <- if (useFDR) -log10(plotData$padj) else -log10(plotData$pval)

  grouped_data <- arrange(
      dplyr::summarize(
          group_by(plotData, pathway),
          avgLogP = mean(logP, na.rm = TRUE)
      ),
      avgLogP
  )

  pathwayOrder <- selectedPathways
  # print(pathwayOrder)
  plotData$dataset <- factor(plotData$dataset, levels = studyIDs)
  plotData$pathway <- factor(plotData$pathway, levels = pathwayOrder)
  yLabels <- pull(arrange(unique(select(plotData, "pathway", sym(yAxis))), as.
↪numeric(pathway)), sym(yAxis))

  scaleMinMax <- function(x, minx, maxx) {
      x[x < minx] <- minx
      x[x > maxx] <- maxx
      x
  }

  if (yAxis == "pathway") {
      plotData$yLabel <- plotData$pathway
  }else {
```

```
        plotData$yLabel <- plotData$name
    }

    plotData$p.value.scaled <- scaleMinMax(abs(log10(
        if (useFDR) plotData$padj else plotData$pval)),
            negLog10pValueLims[1], negLog10pValueLims[2])

    ggplot(plotData, aes(y = .data$pathway, x = factor(.data$dataset))) +
        geom_tile(
            aes(fill = .data$p.value.scaled)
        ) +
        scale_fill_continuous(
            low = "white",
            high = "#CD5C5C",
            limits = c(negLog10pValueLims[1], negLog10pValueLims[2]),
            breaks = c(negLog10pValueLims[1], (negLog10pValueLims[1] +␣
↪negLog10pValueLims[2]) / 2, negLog10pValueLims[2]),
        ) +
        labs(fill = paste0("-log10", ifelse(useFDR, " pFDR", " p-value"))) +

        new_scale_fill() +
        geom_point(
            aes(
                fill = .data$Direction,
                size = .data$abs.NES
            ),
            shape = 21,
            color = "white",
            stroke = 0.5
        ) +
        scale_y_discrete(
            labels = yLabels
        ) +
        scale_size_continuous(
            guide = guide_legend(override.aes = list(shape = 21, fill =␣
↪"gray50"))
        ) +
        scale_fill_manual(
            values = c("Positive" = "#FFAA1D", "Negative" = "#72A0C1"),
            guide = guide_legend(override.aes = list(shape = 21, size = 8),␣
↪title = element_text("Sign"))
        ) +
        labs(
            size = "Normalized score",
            x = "",
            y = "") +
```

```
        theme_bw() +


      ggplot2::theme(plot.title = element_text(hjust = 0.5))
}
```

```
# Rename the columns of the meta-result
colnames(metaResult) <- c("pathway", "NES", "NES.sd", "pval", "padj")
# Create input data for the plotPathwayHeatmap function
PAResults <- list("GSE5281" = gse5281KEGGFGSEA, "GSE153873" =
 ↪gse153873KEGGFGSEA, "Meta-analysis" = metaResult)
# Get description for the top pathways
names(topPathway) <- gsub(" - Homo sapiens \\(human\\)", "",
 ↪KEGGPathways[topPathway])
# Create the heatmap plot
pwHeatmap <- plotPathwayHeatmap(PAResults, yAxis = "name", useFDR = FALSE,
 ↪selectedPathways = topPathway)
pwHeatmap
```

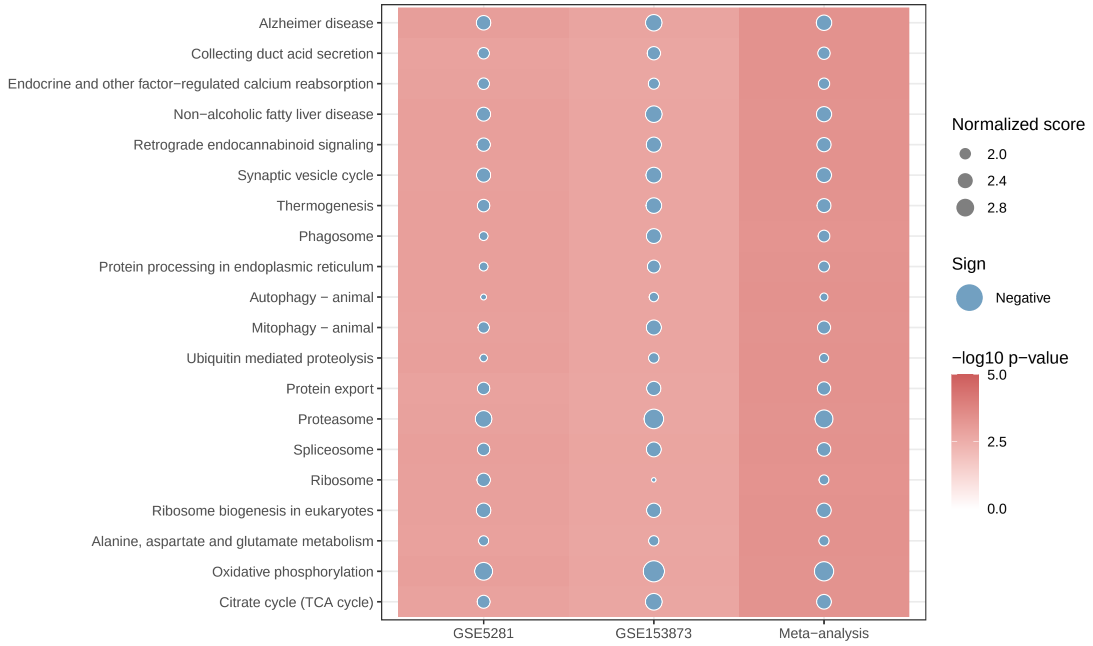

Supplement: Supplementary_Note_bbae222 [file supplementary_note_bbae222.pdf]
